# Chitosan and chito-oligosaccharide: a versatile biopolymer with endless grafting possibilities for multifarious applications

**DOI:** 10.3389/fbioe.2023.1190879

**Published:** 2023-05-19

**Authors:** Popat Mohite, Sunny R. Shah, Sudarshan Singh, Tanavirsing Rajput, Shubham Munde, Nitin Ade, Bhupendra G. Prajapati, Himanshu Paliwal, Dhaval D. Mori, Ashvin V. Dudhrejiya

**Affiliations:** ^1^ AETs St. John Institute of Pharmacy and Research, Palghar, Maharashtra, India; ^2^ B. K. Mody Government Pharmacy College, Gujarat Technological University, Rajkot, India; ^3^ Department of Pharmaceutical Sciences, Faculty of Pharmacy, Chiang Mai University, Chiang Mai, Thailand; ^4^ Shree S. K. Patel College of Pharmaceutical Education and Research, Ganpat University, Kherva, India; ^5^ Drug Delivery System Excellence Centre, Department of Pharmaceutical Technology, Faculty of Pharmaceutical Sciences, Prince of Songkla University, Songkhla, Thailand

**Keywords:** chitosan, chito-oligosaccharides, grafting, drug delivery, wound healing, radical scavenging

## Abstract

Chito-oligosaccharides (COS), derived from chitosan (CH), are attracting increasing attention as drug delivery carriers due to their biocompatibility, biodegradability, and mucoadhesive properties. Grafting, the process of chemically modifying CH/COS by adding side chains, has been used to improve their drug delivery performance by enhancing their stability, targeted delivery, and controlled release. In this review, we aim to provide an in-depth study on the recent advances in the grafting of CH/COS for multifarious applications. Moreover, the various strategies and techniques used for grafting, including chemical modification, enzymatic modification, and physical modification, are elaborated. The properties of grafted CH/COS, such as stability, solubility, and biocompatibility, were reported. Additionally, the review detailed the various applications of grafted CH/COS in drug delivery, including the delivery of small drug molecule, proteins, and RNA interference therapeutics. Furthermore, the effectiveness of grafted CH/COS in improving the pharmacokinetics and pharmacodynamics of drugs was included. Finally, the challenges and limitations associated with the use of grafted CH/COS for drug delivery and outline directions for future research are addressed. The insights provided in this review will be valuable for researchers and drug development professionals interested in the application of grafted CH/COS for multifarious applications.

## Introduction

Chitin (CI) is a naturally occurring polysaccharide made up of repeating units of N-acetylglucosamine (a derivative of glucose). It is one of the most abundant biomaterials on Earth and is found in the exoskeletons of arthropods such as insects, shrimp, and crab, crustaceans such as those of the silkworm, and in the cell walls of fungi (Singh et al., 2021). CI is also found in some species of fish and amphibians. Chemically, CI is a long-chain polymer that forms a crystalline structure, which is composed of chains of N-acetylglucosamine units linked by β-1,4 glycosidic bonds (Eze et al., 2022). It is a hydrophobic material that can be modified chemically to become hydrophilic. It can also be obtained through the cultivation of certain fungi. CI has a wide range of applications; it is used in the production of chitosan (CH), which is known for multifarious applications and used as a water purification agent, wound-healing scaffold, and in the fabrication of drug delivery systems. CI is also used in the development of carriers for bioactive compounds and as a raw material in the production of fertilizers, cosmetics, and food additives. Some of the advantages of CI include its biocompatibility, biodegradability, non-toxicity, its ability to promote tissue growth, and its antimicrobial properties (Nwabor et al., 2020). However, some limitations to CI include the high cost of purification and modification, the difficulty of obtaining large quantities, and the need for further research to fully understand its properties and potential applications. CI has the potential for use in a variety of industries, including the pharmaceutical, agricultural, and biotechnology industries. Specific potential applications include ameliorating the healing of wounds, drug delivery, and as an excipient in tablets and capsules. However, more research is required to fully realize the potential of CI in these fields.

**FIGURE 1:**
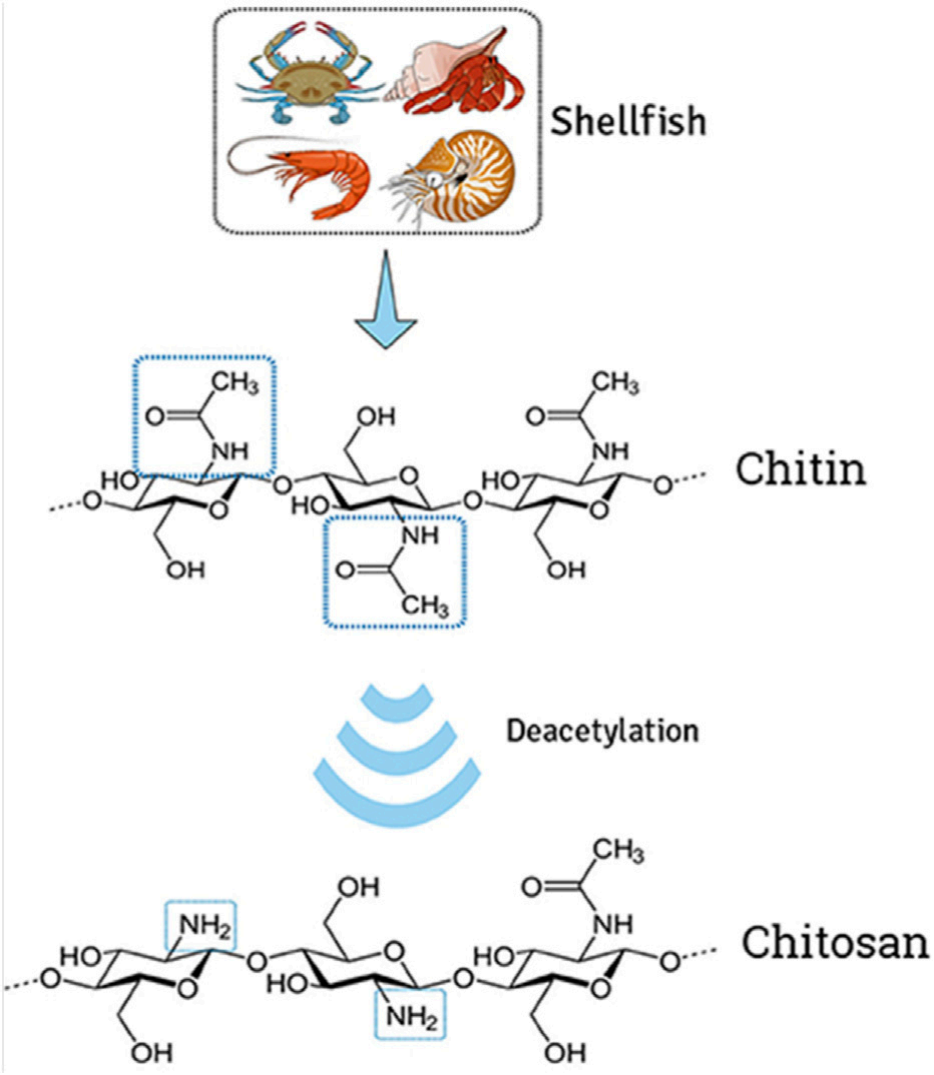
Extraction of chitin from shells and deacetylation to transform chitosan. The illustration demonstrates the modification of natural cationic CH due to the presence of several amino groups composed of β-(1→4) linked D-glucosamine residue, obtained by partial N-deacetylation of chitin. Adapted with permission from Campos et al. (2017) under CC BY version 4.0.

Chitosan (CH) is a derivative of CI, which is a naturally occurring polysaccharide made up of repeating units of N-acetylglucosamine (Figure 1). CH is obtained by the deacetylation of CI, which is typically extracted from the shells of crustaceans such as shrimp and crab. Chemically, chitosan is a polysaccharide composed of randomly distributed β-(1→4)-linked D-glucosamine and N-acetyl-D-glucosamine (Khalaf et al., 2023). It is a cationic polysaccharide, indicating it has a positive charge, which makes it attractive for complexing with negatively charged drugs. The positive charge of CH allows it to bind to negatively charged molecules such as nucleic acids, proteins, and drugs (Wang et al., 2020). This property also makes it an attractive carrier for gene therapy and for delivering drugs to target cells or tissues. Moreover, CH has some advantages as a drug delivery system, including its biocompatibility, biodegradability, non-toxicity, its ability to protect drugs from degradation, and its ability to target specific cells or tissues. CH-based nanoparticles have been investigated for the targeted delivery of anticancer drugs, vaccines, and siRNA (Iacob et al., 2021). However, the limitations associated with CH require further research to completely understand its properties and potential applications.

Chito-oligosaccharides (COS) are short chains of repeating units of N-acetylglucosamine, which is a derivative of glucose, derived from CI and CH. COS are long-chain polysaccharides found in the exoskeletons of arthropods and the cell walls of fungi. COS are generally prepared through various methods such as enzymatic hydrolysis, acid hydrolysis, and chemical oxidation (Charoenpol et al., 2023). Enzymatic hydrolysis is the most commonly used method, which involves the use of chitinases or chitosanases to break down CI or CH into COS (Schmitz et al., 2019). These breakdown products have the advantage of being water-soluble and have a lower viscosity than CH. COS have unique properties that make them interesting for applications in drug delivery, including their lower molecular weight than CI and CH, which makes them more water-soluble. Moreover, COS exhibit a lower degree of acetylation than CH, which makes them more biocompatible. Additionally, COS exhibit lower viscosity and toxicity than CH, which makes them easier to handle and incorporate into various formulations with excellent safety and efficacy. Furthermore, COS present similar properties to CH, which also makes them applicable in drug delivery systems. On the other hand, *Bacillus thuringiensis* (Bt) is one of the most important entomopathogenic agents, which has the ability to form a parasporal crystal comprising Cry proteins that possess insecticidal properties for the control of various species (Bravo et al., 2011). However, in addition to its potential as a biopesticide agent, in recent years, researchers have also studied its ability to degrade chitin and chitosan, based on the secretion of extracellular enzymes as a result of their ecological interactions. It has been observed that Bt possesses chitosanolytic activity, so it is used in the production of COS derived from chitin and chitosan (Camarillo et al., 2004; Ramırez et al., 2004; Ortiz‐Rodríguez et al., 2010; Rosas-García et al., 2013). Rafael et al. compared and explored the production of COS from different sources of CH and found that the process parameter with expanding biotechnology aspects alters the production yields (Olicón-Hernández et al., 2017).

The source of chitosan has a significant impact on its properties and applications. Moreover, both enzymatic and chemical treatments for the step of deproteinization and different conditions of CH preparation significantly alter the degree of acetylation and molecular weight. The different allomorphic forms of chitosan, such as α-, β-, and γ-chitosan, have distinct physical and chemical properties, which make them suitable for different applications (Elieh-Ali-Komi and Hamblin, 2016; Guan et al., 2019). For example, β-chitosan derived from squid pen has higher solubility and reactivity than α-chitosan, making it suitable for the synthesis of thin films and medical, food applications, and biosensors products (Youn et al., 2013). On the other hand, α-chitosan has higher mechanical strength, making it suitable for producing tissue engineering platforms (de Sousa Victor et al., 2020). Chitosan derived from fish has more potential for biomedical and pharmaceutical applications than chitosan of microbial origin (Santos et al., 2020), while α-chitosan derived from marine crustaceans was the first most abundant chitosan readily available in larger amounts from food processing industries, making it broadly used for the production of biomedical products (Hamed et al., 2016).

COS have been investigated for use in various drug delivery applications, such as the targeted delivery of anticancer drugs, gene therapy, and siRNA delivery. They have also been studied for use as excipients in tablet and capsule formulations, and as a coating for implants and medical devices (Raizaday et al., 2022). However, COS are a relatively new and not widely studied biomaterial; more research is required to fully understand their properties and potential applications in drug delivery and to develop effective and safe products based on COS. Therefore, in this review, a wide range of CH and COS applications were considered with endless possibility of grafting.

### Grafting and cross-linking of polymers

Polymer grafting (GRF), a technique that involves the addition of chemical groups or side chains to a polymer backbone, is achieved through various methods, such as chemical modification, radiation-induced grafting, and plasma-induced grafting. The resulting grafted (GRFT) polymer has a new set of properties that can be tailored for specific applications. In the pharmaceutical industry, polymer GRF has shown potential for use in drug delivery systems. For example, GRF of polyethylene glycol (PEG) onto a polymer backbone improves the solubility and stability of drugs (Moretton et al., 2012). Moreover, PEGylation also increases the circulation time of drugs in the body, allowing for targeted delivery to specific cells or tissues. In addition, the GRF of polysaccharides such as CI or CH onto a polymer backbone significantly improves the biocompatibility and biodegradability of the polymer (Bakshi et al., 2020). Furthermore, polymer GRF is also used to modify the surface properties of implants and medical devices, such as increasing their hydrophilicity or increasing their resistance to bacterial adhesion. The chemical modification process is complex and time-consuming and may not be suitable for all types of polymers. Additionally, the properties of GRFT polymers are difficult to predict and control. Overall, polymer GRF is a promising technique with potential applications in the pharmaceutical industry, particularly in drug delivery systems.

GRF and cross-linking (CL) of polymers refer to chemical modifications that alter the physical and chemical properties of polymers, such as their mechanical strength, thermal stability, and biocompatibility. These modifications can be performed through chemical reactions that create covalent bonds between the polymer chains, or by physical means, such as exposure to high temperatures or UV light (Kaczmarek et al., 2020). GRF involves the chemical modification of a polymer backbone through the introduction of new polymer chains, achieved through a variety of techniques, including free radical polymerization, ring-opening polymerization, and living polymerization (Roy et al., 2009). GRF can be used to improve the mechanical properties, thermal stability, and biocompatibility of the polymer (Kai et al., 2018). CL, on the other hand, involves the creation of covalent bonds between polymer chains, resulting in a three-dimensional network structure. CL can be performed through various methods, including chemical, radiation, and physical methods (Gao et al., 2020). CL of polymeric materials is used to improve the mechanical properties, thermal stability, and chemical resistance of the polymer. Recent advances in polymer chemistry have enabled the development of new techniques for GRF and CL, including click chemistry and dynamic covalent chemistry. These techniques allow for the precise control of polymer modification and the creation of highly functionalized polymers with improved properties. In general, GRF and CL are powerful techniques used for the chemical modification of polymers, with the ability to improve the physical and chemical properties of the polymer for specific applications. These modifications have been used in a wide range of fields, including materials science, biotechnology, and biomedical engineering. It is important for researchers and regulatory agencies to have a clear understanding of the implications of polymer modification and the effects on the physical and chemical properties of the polymer.

### Free radical polymerization GRF

Free radical polymerization (FRP) involves the initiation of polymerization with a free radical species and the addition of monomer units to the polymer backbone. FRP is a widely used technique for preparing GRFT polymers to synthesize a wide range of polymers with different properties, including biodegradable polymers, thermo-responsive polymers, and polymer brushes. This method of polymerization is characterized by the initiation of polymerization with a free radical species, which then reacts with monomer units to add new polymer chains to the polymer backbone (Gao et al., 2020). FRP is performed through several methods, including batch polymerization (the reaction of monomers in a closed system with a free radical initiator to produce the polymer), continuous polymerization (continuous addition of monomers to a reactor system with a free radical initiator to produce the polymer), and suspension polymerization (suspension of monomers in a solvent system with a free radical initiator to produce the polymer). The development and characterization of chitosan-graft-poly(N-hydroxyethyl acrylamide) copolymers was reported, where potassium persulfate was used as an initiator to graft poly(N-hydroxyethyl acrylamide) onto chitosan molecules in an aqueous solution. The resulting copolymers exhibited excellent water solubility and biocompatibility, which makes them potential candidates for various biomedical applications, such as drug delivery and tissue engineering (Bahramzadeh et al., 2019). The chitosan-g-(acrylic acid-co-acrylamide) copolymers have been reported to be synthesized through the grafting of chitosan with acrylic acid and acrylamide monomers in the presence of a potassium persulfate free radical initiator. The grafted copolymers exhibited good thermal stability and pH-responsive behavior (Said et al., 2018).

### Ring-opening polymerization GRF

Ring-opening polymerization (ROP) involves the reaction of a cyclic monomer with a nucleophile to produce a linear polymer chain. ROP is a type of polymerization reaction that involves the opening of a cyclic monomer to form a polymer chain (Purohit et al., 2022a) and is used to prepare a wide range of polymers, including biodegradable polymers, hydrogels, and polypeptides (Parkatzidis et al., 2020). Additionally, ROP can be performed in the presence of various initiators, including metal catalysts or organic initiators, to control the rate of polymerization and the molecular weight of the polymer (Brocas et al., 2013; Tschan et al., 2021). This reaction is initiated by the attack of a nucleophile, such as an alkoxide or amine, on the ring structure of the monomer, leading to the formation of a propagating species that can then react with additional monomer units to form a polymer chain. There are several types of ROP including alkene ROP (polymerization of cyclic olefins, such as cyclic esters or cyclic ethers, to form polymers), cyclic amine ROP (polymerization of cyclic amines, such as cyclic imides or cyclic peptides, to form polymers), and cyclic anhydride ROP (polymerization of cyclic anhydrides, such as succinic anhydride or maleic anhydride, to form polymers). A group of researchers developed chitosan-graft-poly(l-lactide) (CS-g-PLLA) copolymers using the “grafting to” approach to create hybrid biomaterials for tissue engineering applications. CS-g-PLLA copolymers have potential as hybrid biomaterials for tissue engineering applications, with their mechanical stability and bioactivity depending on the CS/PLLA ratio (Kaliva et al., 2020). Li et al. (2022) discussed the preparation of biodegradable microspheres using a Schiff base chitosan–polylactide polymer as the encapsulation material for the controlled release of vancomycin, an antibiotic used in bone tissue delivery. The highest drug loading and encapsulation efficiencies were observed for the 1:100 microspheres, and the release rate of the drug was found to be controlled and sustained over a period of 30 days.

### Living polymerization GRF

Living polymerization (LP) involves the initiation of polymerization with a controlled number of active sites, resulting in well-defined polymer chains with a narrow molecular weight distribution (Dey et al., 2021). LP reactions involve the continuous growth of polymer chains, leading to the formation of polymers with well-defined molecular weights and controlled microstructures. This is in contrast to traditional polymerization techniques, such as free radical polymerization or step-growth polymerization, in which the polymer chain is subject to termination reactions that result in a distribution of polymer molecular weights.

There are several types of LP including anionic polymerization (initiation of the polymerization reaction by the abstraction of a leaving group, such as a halide or pseudohalide, from the monomer to form a propagating species that can then react with additional monomer units to form a polymer chain), cationic polymerization (initiation of the polymerization reaction by the protonation of a nucleophile, such as an amine or thiol, to form a propagating species that can then react with additional monomer units to form a polymer chain), and coordination polymerization (initiation of the polymerization reaction by the coordination of a monomer to a metal catalyst to form a propagating species that can then react with additional monomer units to form a polymer chain) (Krishnan et al., 2022). Mahanta and Maiti (2019) reported the development of an injectable hydrogel of CS, which is chemically modified through grafting of ester-diol-based polyurethane to transform into a hydrogel through hydrophilic–hydrophobic balance. Cellular uptake of the drug from the graft copolymer hydrogel has considerably increased compared to a pure drug, resulting in significant enhancement of cell killing using the developed drug-embedded hydrogel system.

### Enzymatic GRF

Enzymatic grafting (EG) involves the use of enzymes to initiate polymerization and add new polymer chains to the polymer backbone. This technique is often used to achieve specific chemical functionalities, such as biodegradability (Shokri et al., 2022). EG is a technique for preparing grafted polymers in which the polymerization reaction is catalyzed by enzymes, specifically, enzymes that catalyze transfer reactions. In enzymatic grafting, a polymer backbone is modified by the transfer of monomer units from a donor molecule to the backbone, typically through the formation of a covalent bond (Choudhary et al., 2020). This reaction is typically performed in the presence of an enzyme that acts as a catalyst, facilitating the transfer of monomer units from the donor molecule to the polymer backbone. There are several types of enzymes that can be used for enzymatic grafting, including transaminases, transglutaminases, and lipases. Transaminases catalyze the transfer of an amine group from a donor molecule to a substrate, while transglutaminases catalyze the formation of covalent bonds between glutamine residues and other amine-containing molecules (Pagar et al., 2021). Lipases catalyze the transfer of fatty acids or other lipophilic groups from a donor molecule to a substrate. Moreover, EG offers several advantages over other methods of preparing grafted polymers, including high selectivity, high efficiency, and low toxicity. Additionally, EG can be performed in mild conditions, avoiding the use of harsh chemicals or elevated temperatures, which can be detrimental to the properties of the grafted polymer.

The synthesis of a polymeric vesicle system using chitosan-g-[peptide-poly-ε-caprolactone] involves functionalizing chitosan with maleimide groups and preparing polycaprolactone with alkyne end-groups. The degradability of the polymersomes is tested using two enzymes, trypsin and chitosanase, and the dispersion of polymersomes is used to coat titanium plates to test the stability against enzymatic degradation (Bourgat et al., 2021). An enzyme-responsive colon-specific drug delivery system is developed using hollow mesoporous silica spheres (HMSS) with biodegradable CS attached via cleavable azo bonds (HMSS–N=N–CS). Doxorubicin loaded in the HMSS and HMSS–N=N–CS showed that they were well-tolerated, and cellular uptake tests showed that DOX uptake increased after pre-incubation with a colonic enzyme mixture (Cai et al., 2020). Li et al. (2021) reported a novel chitinase from *Paenibacillus pabuli* (PpChi) that produces partially acetylated chito-oligosaccharides (paCOSs) with a degree of polymerization (DP) of three and four and a single N-acetylation at their reducing end. PpChi represents an attractive biocatalyst for the green production of highly valuable paCOSs with a well-defined structure.

### Radiation GRF

Radiation grafting (RG) involves the exposure of a polymer to ionizing radiation, such as gamma or electron beam radiation, to initiate free radical polymerization and add new polymer chains to the polymer backbone (Naikwadi et al., 2022). RG is a technique for preparing grafted polymers in which the polymerization reaction is initiated by exposure to ionizing radiation. This technique involves the irradiation of a polymer substrate in the presence of a monomer, which leads to the formation of free radicals on the polymer surface. These free radicals then react with the monomer to form a grafted polymer. RG is performed using a range of ionizing radiation techniques including gamma rays, electrons, and X-rays. The choice of ionizing radiation depends on the properties of the polymer and monomer and the desired properties of the grafted polymer. One advantage of RG is that it allows for the preparation of GRFT polymers with high molecular weight and high grafting efficiency. Additionally, radiation grafting can be performed in the absence of solvents or other chemical initiators, which can simplify the synthesis process and reduce the cost of production.

Kumar et al. (2019) reported the synthesis and characterization of antimicrobial grafted chitosan as a drug delivery device for the controlled release of curcumin drug using a microwave procedure. The antimicrobial activity of the grafted chitosan against *Escherichia coli* and *Staphylococcus aureus* was found to be superior to un-grafted chitosan. The authors reported the synthesis of a novel antibacterial copolymer of chitosan using acrylic acid and acrylonitrile as monomers and a microwave-assisted method. The copolymer exhibited excellent antimicrobial activity against both Gram-negative and Gram-positive bacteria (Kumar et al., 2018). CS-g-maleic acid is a type of modified chitosan that is prepared using gamma radiation, which involves the grafting of maleic acid onto the chitosan backbone. This modification enhances the adsorption properties of chitosan by increasing the number of functional groups available for binding metal ions (Zhuang et al., 2018).

### Photochemical GRF

Photochemical grafting (PG) involves the use of light-activated initiators to initiate polymerization and add new polymer chains to the polymer backbone. This technique is often used for the synthesis of photoresponsive polymers, which can be used for applications such as drug delivery (Michalek et al., 2020). PG is a technique for preparing grafted polymers that involves the initiation of the polymerization reaction through exposure to light, which involves the irradiation of a polymer substrate in the presence of a monomer and a photo-initiator that triggers the formation of free radicals on the polymer surface. These free radicals then react with the monomer to form a grafted polymer. One advantage of PG is that it allows for precise control over the initiation and rate of the polymerization reaction, as well as the spatial distribution of the grafted polymer. Additionally, PG can be performed in aqueous or organic solvents and can be used to prepare grafted polymers with a variety of functional groups. CS particulate hydrogels (CPH) developed and modified through the Michael addition of poly(ethylene glycol) methyl ether acrylate (PEGA) to the amine groups were subjected to double crosslinking, ionic and covalent, in water/oil emulsion. CPH had excellent capacity for drug loading and controlled release potential along with cyto- and hemocompatibility, which is suitable for different biomedical applications (Logigan et al., 2022). The adhesiveness and growth of human umbilical vein endothelial cells (ECs) can be improved by photochemically grafting the cell-adhesive peptide Gly–Arg–Gly–Asp (GRGD) on chitosan and chitosan–tripolyphosphate (TPP) surfaces. The human umbilical vein ECs adhered and grew well on chitosan-GRGD and chitosan-TPP–GRGD surfaces after 36 h of incubation, and the viability of the cells on these surfaces was confirmed by MTT assay (Chung et al., 2002).

### Chemical CL

Chemical CL is a technique for preparing GRFT polymers, which involves the formation of covalent bonds between the polymer chains. This is typically accomplished through the addition of a CL agent that reacts with the functional groups present on the polymer chains to form covalent bonds. Chemical CL is used to prepare GRFT polymers with a variety of properties, including improved mechanical strength, thermal stability, and biocompatibility. For example, chemical cross-linking can be used to prepare grafted hydrogels for use in biomedical applications, such as drug delivery and tissue engineering (Ahmad et al., 2022). Chemical cross-linking was employed to prepare hydrogels from starch and chitosan, involving the synthesis of a starch precursor through carboxymethylation using sodium monochloroacetate, followed by mixing of the modified starch with chitosan. Methacrylic acid was used as a cross-linker to graft the chitosan onto the starch chains. The combination of methacrylic acid and carboxymethyl starch (CMS)–chitosan mixture resulted in the highest grafting yield, grafting efficiency, and monomer conversion (Sunarti et al., 2019). Georgopoulou et al. (2018) evaluated a chitosan-graft-polycaprolactone (CS-g-PCL) copolymer as a potential biomaterial for bone tissue engineering. They observed that MC3T3-E1 pre-osteoblastic cells adhered strongly to the copolymer surfaces from the first days of cell culture and had a characteristic spindle-shaped morphology. The enhanced osteogenic response of the MC3T3-E1 pre-osteoblasts cultured on the CS-g-PCL copolymer suggests that it is a promising candidate for bone tissue engineering. The copolymer supports cell functions toward bone tissue formation and can potentially be used for the development of new bone substitutes or implants.

### Radiation CL

Radiation CL is another technique for preparing GRFT polymers that involves the formation of covalent bonds between the polymer chains through exposure to high-energy radiation (Ahmad et al., 2022). This process can be performed using many types of radiation, including ultraviolet radiation, gamma radiation, and electron beam radiation. One advantage of radiation CL is that it can be performed at room temperature and pressure, which makes it an attractive alternative to chemical cross-linking in certain applications. Radiation cross-linking has been widely used to prepare grafted polymers for a variety of applications, including drug delivery, tissue engineering, and wound healing. For example, grafted hydrogels prepared by radiation cross-linking have been used for the controlled release of therapeutic agents, such as growth factors and small molecule drugs. Additionally, new methods for inducing cross-linking in the presence of nanoparticles and for controlling the mechanical properties of grafted polymers have been developed (Qamruzzaman et al., 2022). Tan et al. (2021) synthesized a diclofenac-loaded hydrogel from carboxymethyl sago pulp (CMSP) and chitosan by electron beam (EB) irradiation to impart pH- and temperature-sensitive swelling behavior. The 40% CMSP/3% chitosan hydrogel was found to be a potential drug carrier for sustained drug delivery, along with adequate antimicrobial activity against tested microorganisms. The binary graft of N-vinylcaprolactam (NVCL) and pH-sensitive acrylic acid (AAc) monomers onto chitosan gels (net-CS) was reported to be fabricated through pre-oxidative irradiation grafting and direct grafting methods. The study showed that the binary graft of chitosan gels depicted thermo- and pH-sensitive release behavior (Ortega et al., 2021).

### Physical CL

Physical CL is a technique used to prepare graft polymers by forming covalent bonds between polymer chains through physical processes. Physical CL is achieved through a variety of methods including thermal cross-linking that occurs through the application of heat, and mechanical cross-linking that occurs through mechanical force (Yang et al., 2022a). Other methods of physical CL include solvent-induced CL and ultrasound-induced cross-linking. Physical CL offers several advantages, for example, it does not require the use of chemical reagents, and is, therefore, a more environmentally friendly approach. Additionally, physical CL can be performed at room temperature, making it less expensive and less energy-intensive than other CL methods. The chitosan/κ-carrageenan hydrogel prepared by Mahdavinia et al. (2017) was reported to exhibit potential applications in drug delivery, wound healing, and tissue engineering. The hydrogel’s properties, such as its swelling behavior, mechanical strength, and degradation rate, can be tailored by adjusting the composition of the hydrogel and the conditions of preparation. These natural polymers are biocompatible, biodegradable, and non-toxic, and readily available and relatively inexpensive compared to synthetic polymers. Wang et al. used a ternary solvent system to prepare pure chitosan films with a micro-nanostructure, which is a novel approach to improving the water uptake capacity of chitosan films. The outcome of the study highlighted the potential for optimizing the physical microstructure of chitosan films to improve their water absorbency, which could have important implications for various applications in the fields of biomedicine and hygienic products (Wang et al., 2016).

### Characterization of grafted polymers

Polymer GRF improves morphology, chemistry, and physics, with improvement in polymer conduction and features other than charge transport, improving solubility, nano-dimensional shape, biocompatibility, bio-communication, and other qualities (Purohit et al., 2022b). Different analytical techniques are employed for the characterization of GRFT polymers, such as infrared spectroscopy (FTIR), X-ray diffraction (XRD), scanning electron microscopy (SEM), and differential scanning calorimetry (Bundschuh et al., 2018) and microscopy (Vega-Hernández et al., 2021). FTIR is performed to characterize the presence of specific functional groups blended with grafted polymers. X-ray powder diffraction of a material with the GRFT polymer was recorded using an X-ray diffractometer to analyze the crystal structure, crystal size, and strain. The surface morphology of GRFT polymers is examined using SEM/FESEM equipment. The thermal property of the GRFT polymer is quantified using TGA/DSC/DTA to obtain the purity and melting point with the thermograms of materials.

**FIGURE 2:**
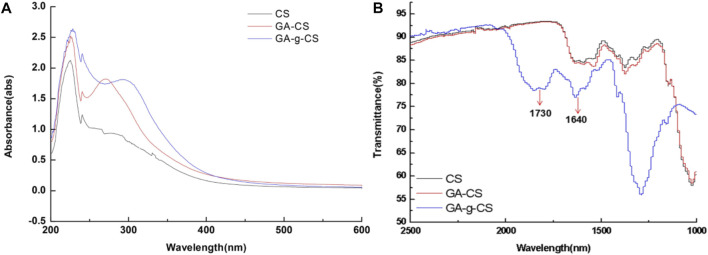
The formation of covalent bonds between chitosan (CS), the physical mixture of gallic acid with chitosan (CS), and enzymatic grafted gallic acid with chitosan was observed using UV-vis spectra **(A)**. Both CS and gallic acid showed absorption peaks between 250 and 350 nm. Meanwhile, in the spectra of gallic acid, GRFT CS exhibited an absorption peak at 262 nm shifted from 272 nm, compared to that of the gallic acid CS physical mixture attributed to the decrease in energy required for π-π* transition, due to covalent linkage of gallic acid and CS. FTIR spectra of chitosan (CS), physical mixture of gallic acid with chitosan (CS), and enzymatic grafted gallic acid with chitosan **(B)**. The FTIR spectra indicated that between 4,000 and 2,500 cm^−1^ and 1,000–400 cm^−1^ for tested samples were identical. However, a slight shift was observed around 1,730 and 1,640 cm^−1^, which indicated the presence of C=O stretching in esters and -C=O stretching of CS amide. Adapted with permission from Zheng et al. (2018) under CC BY version 4.0.

**FIGURE 3:**
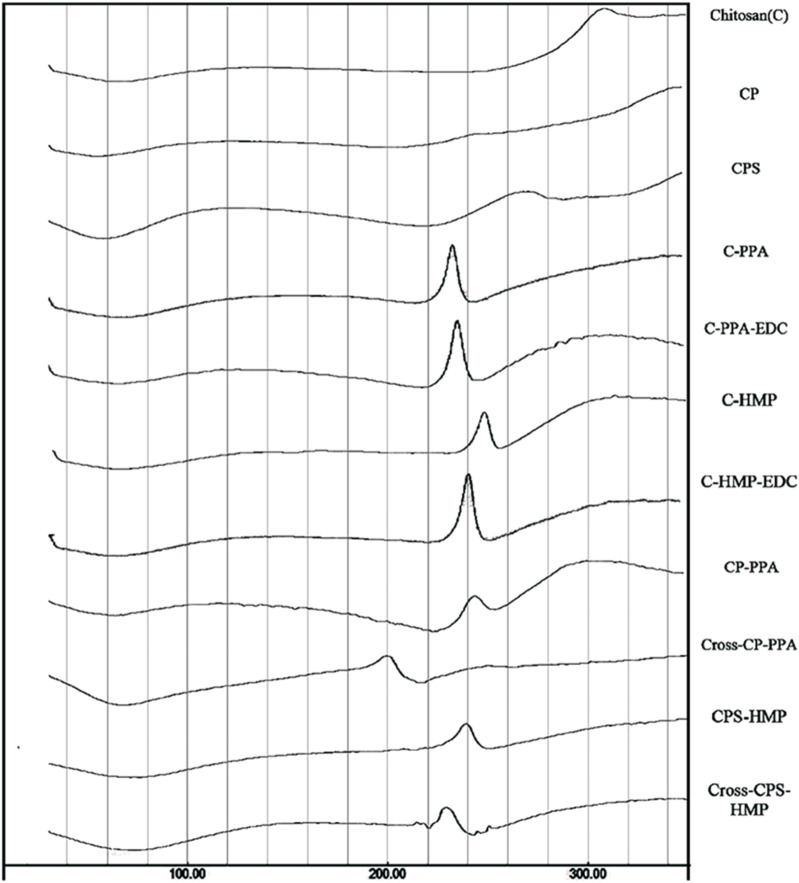
Thermogram (DSC) of unmodified chitosan (C), grafted chitosan phthalate (CP), grafted chitosan phenylsuccinate (CPS), and their ionotropic/covalent corresponding nanoparticles. Chitosan showed typical polysaccharide thermal traits characterized with two bands. The first is an endothermic wide band that extends from 40°C to 100°C, corresponding to polymeric dehydration. The second thermal event is an exothermic band extending from 280°C to 320°C, corresponding to polymeric degradation. CL C showed a shallow exothermic band extending from 220°C to 274°C, attributed to thermally mediated amide-forming reactions linking phenylsuccinic acid moieties and the adjacent C amine group in the modified CP. Moreover, probed ionotropic nanoparticles demonstrated intriguing exothermic peaks, resulting from certain heating-induced exothermic incidents within nanoparticles within the matrix. Adapted with permission from Saeed et al. (2020) under CC BY version 4.0.

**FIGURE 4:**
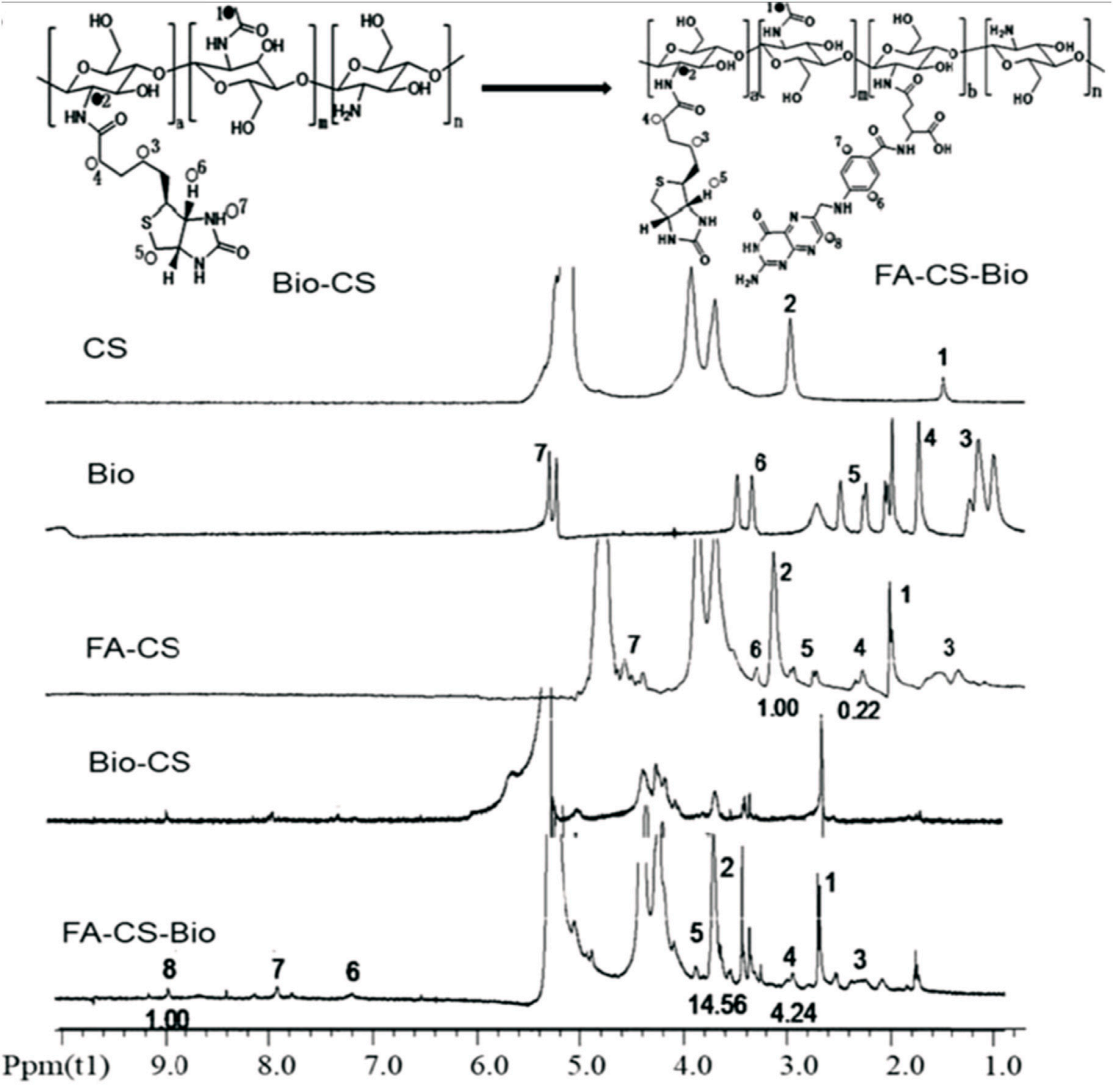
NMR spectroscopy of chitosan (CS), biotin (Bio), folic acid-grafted chitosan (FA-CS), biotin-modified chitosan (Bio-CS), and folic acid-grafted biotinylated chitosan (FA-CS-Bio). The vibration peaks at 1 and 2 on the CS nuclear magnetic spectrum correspond to the protons in its molecular structure for Bio, 3 (4.99 ppm), 4 (5.69 ppm), 5 (6.60 ppm), 6 (7.63 ppm), and 7 (9.98 ppm), were the vibrational peaks of protons in the molecular structure, respectively, and the vibrational peak at 8 (15.63 ppm) attributed to the carboxyl proton hydrogen at the end of the five-carbon chain. Furthermore, the NMR peaks of the synthesized Bio-Cs indicated successful GRFT of CS with Bio, indicated by characteristic peaks 1 and 2 on the FA-CS-Bio hydrogen spectrum, which were assigned to the corresponding protons in CS, of which the integral area of 1 was 14.56, and the characteristic peaks 3, 4, and 5 assigned to Bio molecules, of which the integral area of 4 was 4.24. Moreover, a DS of 29.12% (4.24/14.56) indicated that the CS was coupled with Bio. Additionally, a DS of 6.8% (1/14.56) demonstrated that the synthesized material CS-Bio was coupled with FA to generate FA-CS-Bio. Adapted with permission from Cheng and Dai (2022) under CC BY version 4.0.

**FIGURE 5:**
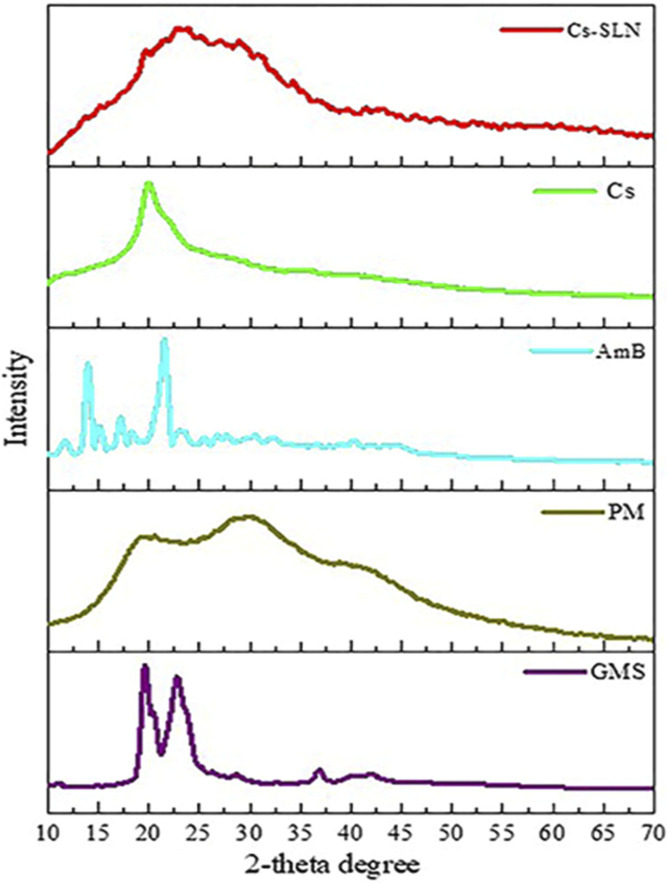
XRD spectra of glycerol monostearate (GMS), amphotericin (AmB), paromomycin, chitosan (Cs), and grafted chitosan-functionalized solid lipid nanoparticles (SLN) of drug (Cs-SLN). X-ray diffractogram of AmB exhibited a sharp peak at the 2θ scattered angle of 21.39, 14.1, and 21.78°, indicating its crystallinity. However, PM was found to be amorphous in nature. Cs-SLN showed two peaks at 24.8° and 30.6°. Nonetheless, the pattern was shifted and broadened, and a weaker peak than GMS was partially recrystallized and transformed to less order in SLN formulation. GMS peaks were also absent due to the complete entrapment of the drug in the lipid matrix. The broadening of the diffraction peaks was attributed to particle sizes as the broadening of Bragg’s peaks indicates the formation of nanoparticles. Adapted with permission from Parvez et al. (2020) under CC BY version 4.0.

In this review, the necessary details from studies are gathered to demonstrate the characterization GRFT-CH/COS. Several studies on the characterization of GRFT-CH/COS have been reported. The UV-vis and FTIR spectra of unmodified and modified CH are shown in Figure 2, indicating a shift in peaks after GRFT, compared with unmodified CH. Thermal analysis using DSC is shown in Figure 3, demonstrating a change in crystallinity. Figure 4 presents the NMR spectra of unmodified and modified CH with an active constituent, GRFT-CH-folic acid, which indicated the presence of several additional peaks, compared to CH alone. The XRD spectra of GRFT CH corroborated with the results of thermal analysis with an alteration in crystallinity are shown in Figure 5. Table 1 describes various methods used to analyze grafted polymers, including XRD, SEM, and particle size as characterization techniques. Furthermore, patented information on GRFT-CH/COS is given in Table 2.

**TABLE 1:** Recent reports on various grafted chitosan and chito-oligosaccharides.

Polysaccharide	Graft chain	Technique	Property measured	Reference
CH	Silver	SEM	The external structure morphology of CH silver nanoparticles was determined by SEM	Dara et al. (2020)
		XRD	XRD at 2θ with a 10–80 fixed time mode was used to capture XRD patterns of the sample	
CH	Phenolic acid	DSC	Thermal property of CH-GRFT phenolic acids was investigated by DSC	Yang et al. (2022b)
		SEM	Microstructure of CH-GRFT phenolic acids was established by SEM	
CH	Catechol	SEM	Structural morphologies of the films were tested by SEM	Cao et al. (2020)
		XRD	States of matter were characterized by X-ray diffraction	
CH	Quercetin	DSC	Thermal property of CH-GRFT quercetin was investigated by DSC	Diao et al. (2020)
		XRD	States of matter of CH-GRFT quercetin were characterized by X-ray diffraction	
CH	Acrylamide	DSC	Thermal property of CH-GRFT acrylamide was investigated by DSC	Geeva Prasanth et al. (2019)
		SEM	Morphology of the films was tested by SEM	

**TABLE 2:** Patented grafted chitosan and chito-oligosaccharide-grafted polymers.

Patent title	Functional group grafted	Patent number	Year	Reference
PEGylated and fatty acid GRFT COS, synthesis method, and application for drug delivery systems	PEG and fatty acid	US 8,466,127 B2	2013	Raizaday et al. (2022)
Amine GRFT CH nanofiber, a method for preparation thereof, and its use in heavy metal adsorption	Amino group	US 9.289,746 B2	2016	Moretton et al. (2012)
Sulfhydrylated cholesterol modified mPEG GRFT CH, preparation method, and application thereof	mPEG	CN111499874B	2020	Bakshi et al. (2020)
Phenylboronic acid GRFT COS derivative, preparation method, and application thereof	Phenylboronic acid	CN113754793A	2020	Kaczmarek et al. (2020)
Phenolic acid GRFT-COS, preparation method, and application of modified waterborne polyurethane of phenolic acid GRFT-COS	Phenolic acid	CN108676108B	2018	Roy et al. (2009)
Preparation method of a cyclodextrin GRFT CH polymer with water pollutant-separating property	Cyclodextrin	CN104530437A	2014	Kai et al. (2018)
A kind of GRFT CH bleeding-stopping dressing with a three-dimensional multi-network and preparation method thereof	Glycerol	CN104258451B	2014	Gao et al. (2020)
Hypromellose GRFT CH and methods of use thereof for sustained drug delivery	Hypromellose	CN107921002B	2016	Purohit et al. (2022a)
Glycolic acid GRFT CH copolymer, preparation method thereof, and application of glycolic acid GRFT CH copolymer used as a scleral buckling material	Glycolic acid	CN102516412B	2011	Parkatzidis et al. (2020)
A kind of partially GRFT COS surfactant and preparation method	N-oleoyl glutamine	CN107081110A	2017	Brocas et al. (2013)

### Biological efficacy and application of grafted CH and COS

**FIGURE 6:**
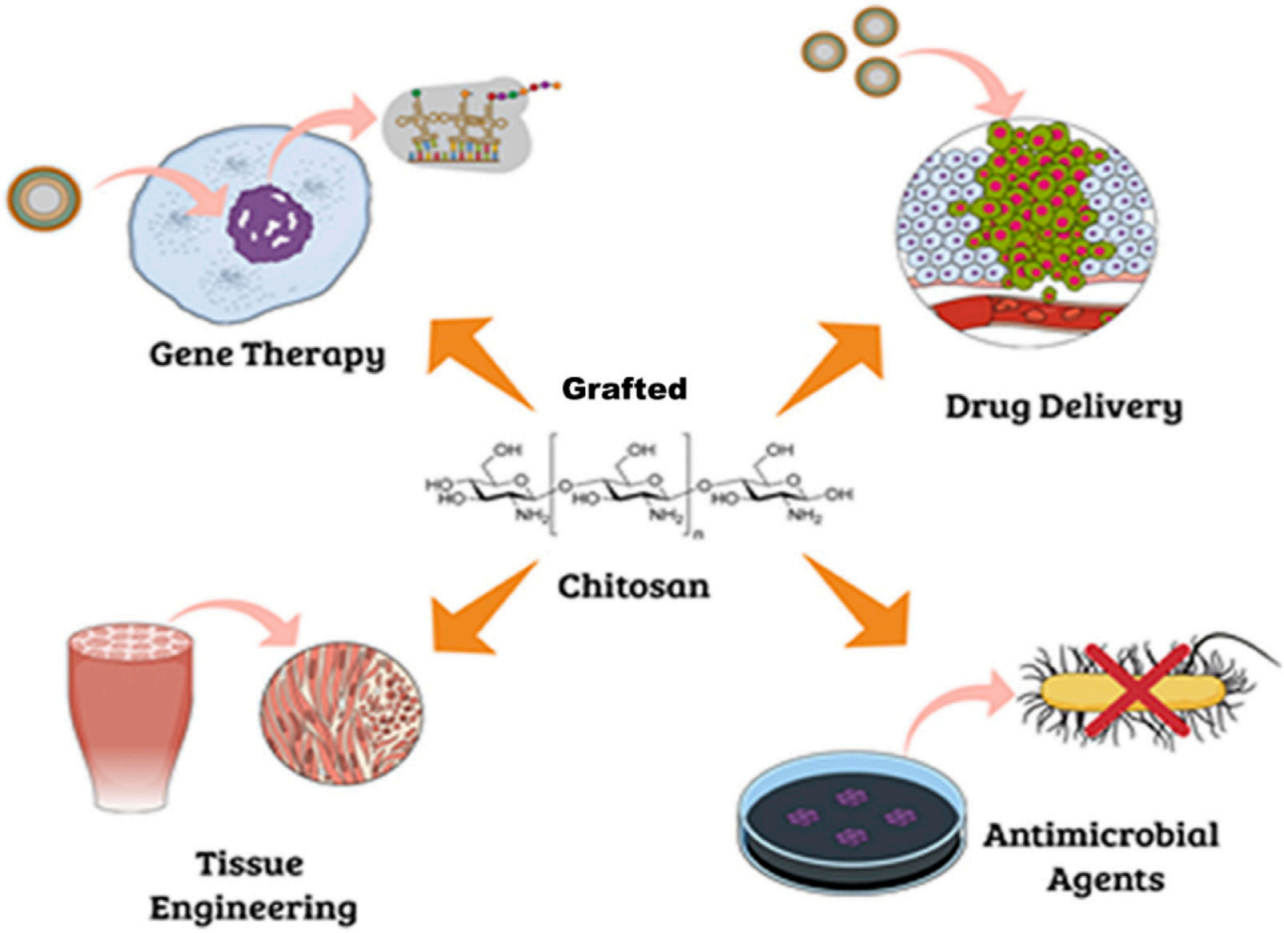
Potential biological application of grafted chitosan. Adapted with permission from Campos et al. (2017) under CC BY version 4.0.

CH and biodegradable polymers can be CL or GRFT directly since their functional groups allow the reaction with the help of heat, vacuum, a catalyst (such as transglutaminase or tyrosinase), or a suitable medium (Sanchez-Salvador et al., 2021). However, if the degree of polymerization of a CH > 20 and its average molecular weight >3,900 g/moL, the polymer identified as COS. COS have unique biological capabilities and are more soluble in a wider range of pH values and at higher concentrations than their counterpart, CH. CH, in its native form, has limited solubility and low mechanical strength, which can be improved by introducing side chains through GRF. However, COS dissolve in a wide variety of solvents, including water/alcohol solutions, DMSO, and DMF, depending on their size. Hence, researchers are focusing on ways to tailor these oligomers to make them useful in more contexts (Chapelle et al., 2021). GRFT-CH, a biocompatible and biodegradable polymer derived from CI, has attracted significant attention in various fields, including drug delivery, food preservation, and in allied science. GRF-CH has been achieved using various techniques, such as chemical modification, enzymatic modification, and physical modification (Choudhary et al., 2020; Pagar et al., 2021). The resulting GRFT-CH exhibits improved solubility, mechanical strength, and biocompatibility, which are critical for its multifarious applications (Figure 6).

The unsatisfactory solubility of CH has been a key barrier to its modification and application. To improve its solubility, a novel organo-soluble glycidol CH was developed as a potential material for bio-related application. The improved organo-soluble glycidol was further explored as an amphiphilic glycidol CH-GRF polycaprolactone copolymer as an efficient carrier of antitumor doxorubicin (Zhang and Cao, 2014). CH-GRF-polyvinyl alcohol copolymers were assessed for oral drug delivery. CH-GRF-polyvinyl alcohol (PVA) indicated excellent hydrogel-forming capacity, which enhanced the bioavailability of drugs when taken orally, reducing the amount of the drug that is metabolized before it reaches the site of action (Li et al., 2021). CH-GRF dextran copolymers were used as a carrier for drugs that are administered intravenously, allowing for improved circulation and reducing the risk of toxicity (Naikwadi et al., 2022). CH-GRF-poly(lactide-co-glycolide) (PLGA) copolymers are highly effective in the fabrication of controlled drug release. PLGA is a biodegradable polymer that can be used to control the release of drugs over an extended period, providing sustained therapy and reducing the frequency of dosing (Makadia and Siegel, 2011). Moreover, COS have been used as carriers for various therapeutic agents. For instance, COS have been successfully GRFT with various anticancer drugs, resulting in improved therapeutic efficacy and reduced toxicity (Kumar et al., 2019). The rationale behind the use of COS is that they can provide a biocompatible, biodegradable, and non-toxic environment for the delivery of drugs. Additionally, the presence of multiple hydroxyl groups in COS provides ample sites for chemical modification, making it an ideal scaffold for drug conjugation. For example, COS have been used as a carrier for the targeted delivery of anticancer drugs, such as paclitaxel and doxorubicin (Kumar et al., 2018). The drug-loaded COS easily attached to the targeted tumor cells via specific ligands, increasing the drug concentration in the tumor site and reducing the systemic toxicity of the drug. In gene therapy, COS have been investigated as a carrier for gene therapy. The small size of COS allows for the efficient encapsulation of plasmid DNA, protecting it from degradation and improving its uptake by target cells (Zhuang et al., 2018). In addition, COS demonstrate improved stability of the plasmid DNA in the bloodstream and enhance its transfection efficiency (Michalek et al., 2020). Furthermore, COS have been used as a delivery system for insulin, a hormone that regulates blood sugar levels in patients with diabetes. The mucoadhesive properties of COS allow prolonged residence time at the site of administration, increasing the bioavailability of insulin and reducing the need for frequent injections (Wong, 2009). Moreover, COS have been used as a carrier for antiviral drugs, such as ribavirin, to treat viral infections (Lembo et al., 2018).

### Food preservation efficacy of GRFT CH or COS

In food preservation, alone and GRFT-CH or COS demonstrated improved antifungal and antimicrobial activity. For example, CH-based edible films and coatings have been developed to extend the shelf life of fresh meats, dairy products, and baked goods (Chung et al., 2002). These films help maintain the quality and freshness of the food products by controlling the water vapor permeability and limiting the growth of bacteria and fungi. CH and COS are investigated to prevent spoilage in stored grains by controlling insect pests and reducing the growth of molds. CH-based microencapsulation has been used to preserve the flavor, color, and aroma of food ingredients such as spices, herbs, and essential oils, whereas COS have been used to extend the shelf life of packed food products such as sausage, ham, beef, bread, cakes, and pastries as well. Additionally, CH-based nanoparticles have been used as a delivery system for natural antimicrobial compounds, such as essential oils and bacteriocins, to increase their efficacy and stability in food preservation, while COS can be incorporated into sea food and meat products in the form of coatings, sprays, or injections to prevent the growth of bacteria and mold. The positively charged nature of COS makes them more effective in inhibiting the growth of a variety of microorganisms, including bacteria and fungi (Ahmad et al., 2022) and makes them a more suitable alternative to traditional chemical preservatives. The adhesive properties of COS allow for improved adherence to the surface of the produce, reducing water loss and maintaining the freshness of the produce (Sunarti et al., 2019). Overall, GFRT-CH/COS have demonstrated their potential as a versatile material for various applications, including drug delivery and food preservation, and are a promising area for further research and development.

### Antimicrobial activity of GRFT CH or COS

**FIGURE 7:**
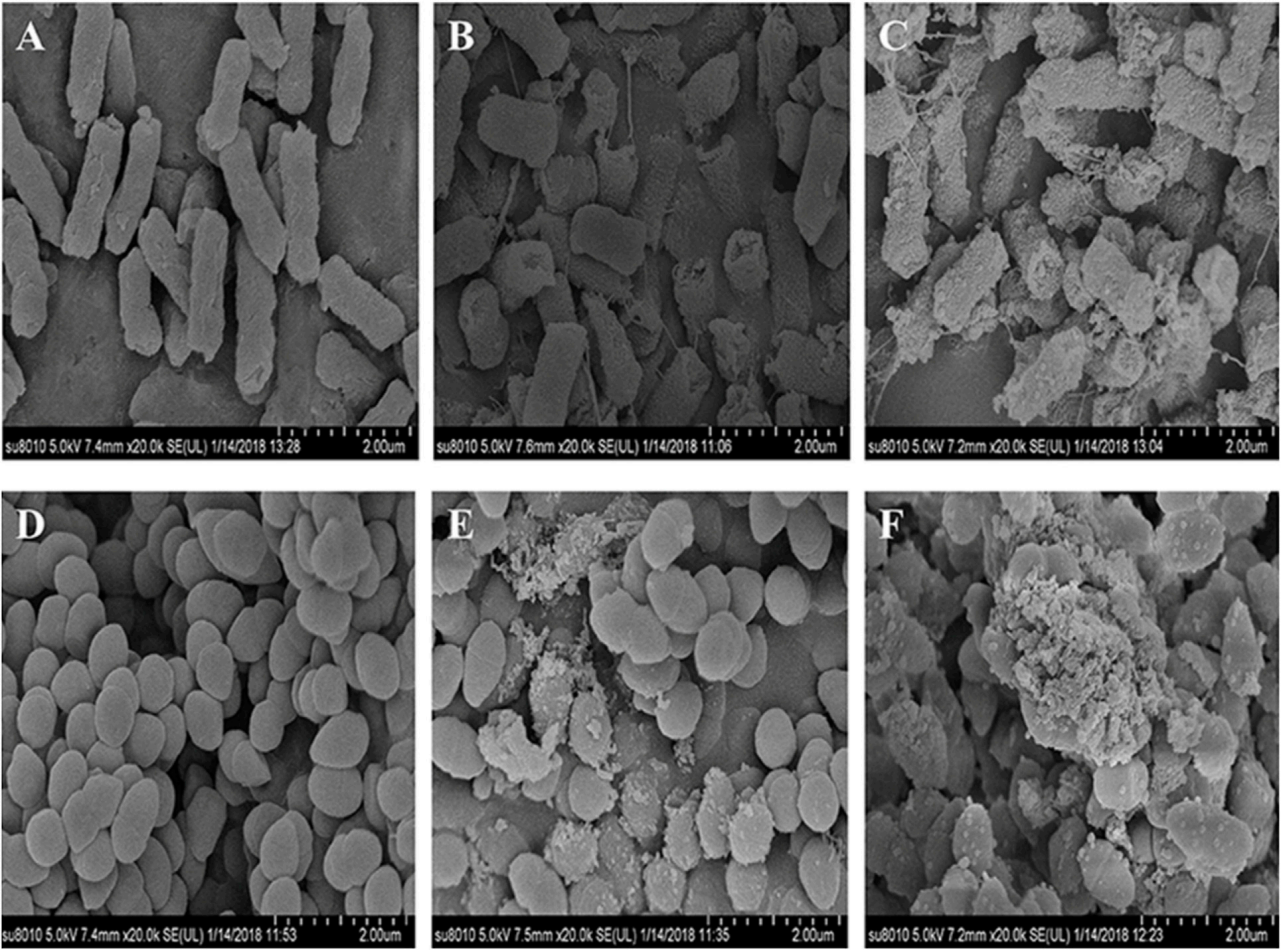
SEM micrograph of *E. coli* and *S. xylosus* **(A, D)**, treated with 10 mg/mL CD-gCS **(B, E)** and 10 mg/mL CD-g-CS **(C, F)**. The control *E. coli* and *S. xylosus* cell membranes with electron-dense lines. On the other hand, *E. coli* treated with CD-g-CS showed a disrupted and altered cell membrane after 1.5 h, especially in the groups treated with CD-g-CS. The CD-g-CS-treated *E. coli* cells exhibited extracellular and intracellular changes compared to untreated cells. These changes might be due to the disruption of outer membrane structures with membrane sloughing and breaching, formation of irregular cell shapes and morphologies, degradation of bacilliform cells into short rods, formation of irregular condensed masses with bleb-like shapes, development of faint bacterial profiles as a result of the loss of cell contents, blurring of the cell surface, and the development of dim, hollow, and even crushed structures. Adapted with permission from Ding et al. (2019) under CC BY version 4.0.

**FIGURE 8:**
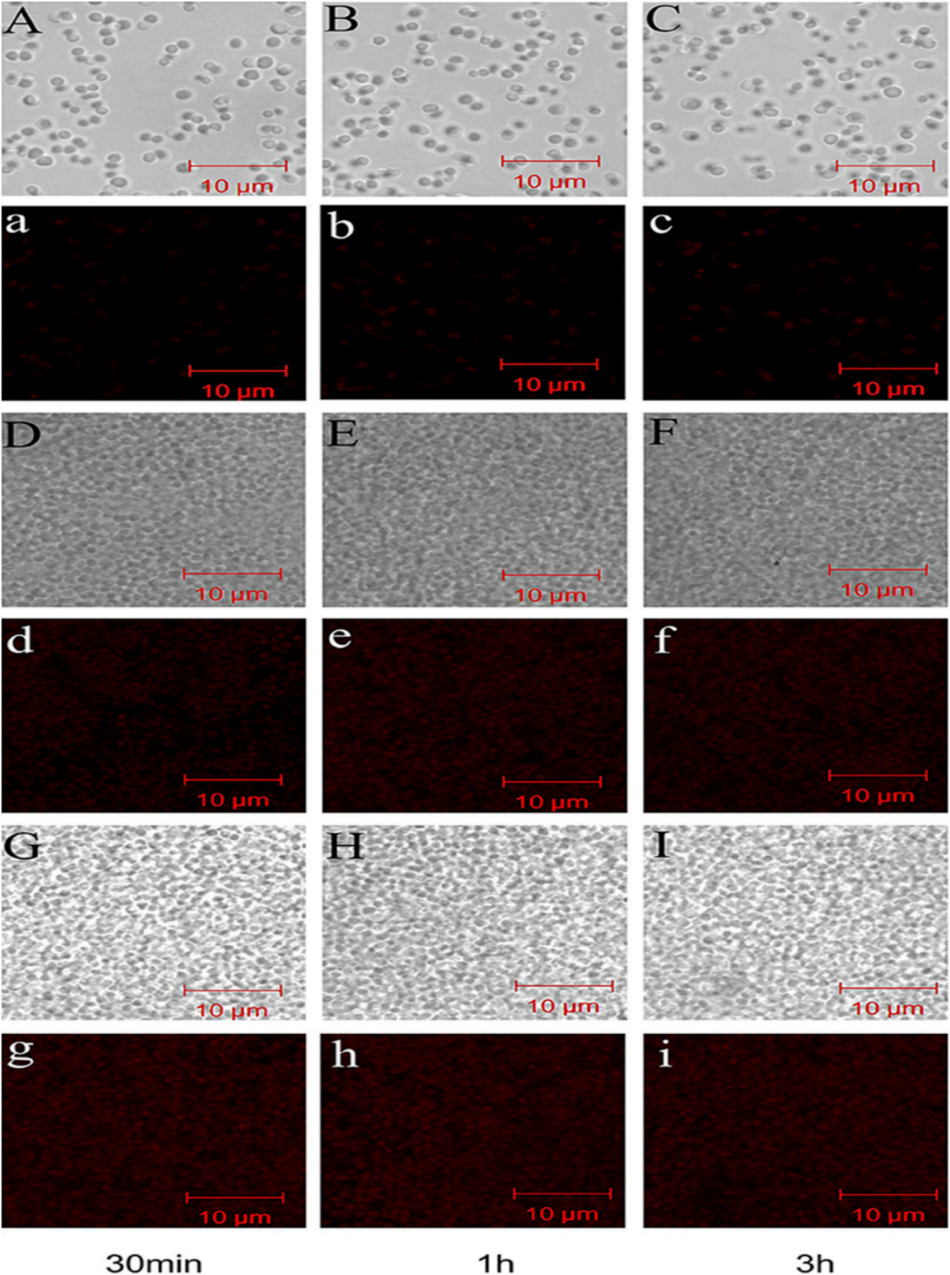
Confocal microscopy image showing the uptake of doxorubicin hydrochloride in *S. xylosus*. **(A–C)** Image of *S. xylosus* treated with only doxorubicin hydrochloride for 30 min, 1 h, and 3 h, respectively. **(D–I)** *S. xylosus* treated with CD-g-CS at different concentrations containing doxorubicin hydrochloride for 30 min, 1 h, and 3 h, respectively. **(A–I)** Bright field CLSM image. The fluorescence intensities of *S. xylosus* treated with doxorubicin hydrochloride alone for 30 min, 1 h, and 3 h were very weak, with no significant differences. However, on increasing the incubation time for the same concentrations of CD-g-CS, the fluorescence of *S. xylosus* was enhanced. Moreover, the fluorescence intensity was similar after *S. xylosus* treated with CD-g-CS (QCD = 0.643 × 10^3^ μmol/g) containing doxorubicin hydrochloride was incubated for 30 min, 1 h, and 3 h, respectively. Similar results were observed for the CD-g-CS-altered group, compared to the CD-g-CS (QCD = 0.643 × 10^3^ μmol/g) groups; the fluorescence intensity was higher in the CD-g-CS (QCD = 0.6 × 10^2^ μmol/g) groups. Adapted with permission from Ding et al. (2019) under CC BY version 4.0.

Several investigations have demonstrated that CH exhibits excellent antibacterial activity (Yilmaz Atay et al., 2019). As pH of the surrounding environment becomes more acidic, positively charged CH reacts with negatively charged residues of biomolecules on the bacterial cell wall, inhibiting bacterial growth (Fuster et al., 2020). Allan and Hadwiger first reported the antifungal activity of CH (Guan and Feng, 2022). Several studies confirm its potent antimicrobial activity against Gram-positive and Gram-negative bacteria and fungi as CH shows the presence of an -NH^+3^ group that binds to the negatively charged membrane components of pathogens (peptidoglycans or O-antigen of lipopolysaccharides) (Tachaboonyakiat, 2017). Younes et al. reported that a lower extent of CH acetylation and lower pH favor better antibacterial activity. Mostafa et al. investigated the potential COS as an alternative for single or in combination with antibiotic treatment. The results indicated a significant impact on the growth of *Streptococcus agalactiae* and *S. aureus* biofilms with an indication to combat antimicrobial resistance (Abd El-Hack et al., 2020). Younes et al. reported that a lower extent of CH acetylation favors improvement in antibacterial activity. Mycelial growth of *Microsporum canis* was inhibited by 100.00% and 93.13% by the nanogels fabricated using chitosan-g-3,4-methylenedioxycinnamic acid-carvacrol and chitosan-g-3,4-methylenedioxycinnamic acid-thymol, respectively (Ladeira et al., 2023). CH-GRFT with ß-cyclodextrin at different concentrations (Q_CD_: 0.643 × 10^3^ and 0.6 × 10^2^ μmol/g) demonstrated significant antimicrobial activity with a loss of membrane activity against *E. coli* and *Scaphopoda xylosus* (Figure 7). Moreover, the confocal image showed the uptake of doxorubicin conjugated with CH-GRFT with ß-cyclodextrin in *S. xylosus* at different time intervals (Figure 8) (Ding et al., 2019).

### Free radical scavenging activity of GRFT CH or COS

**FIGURE 9:**
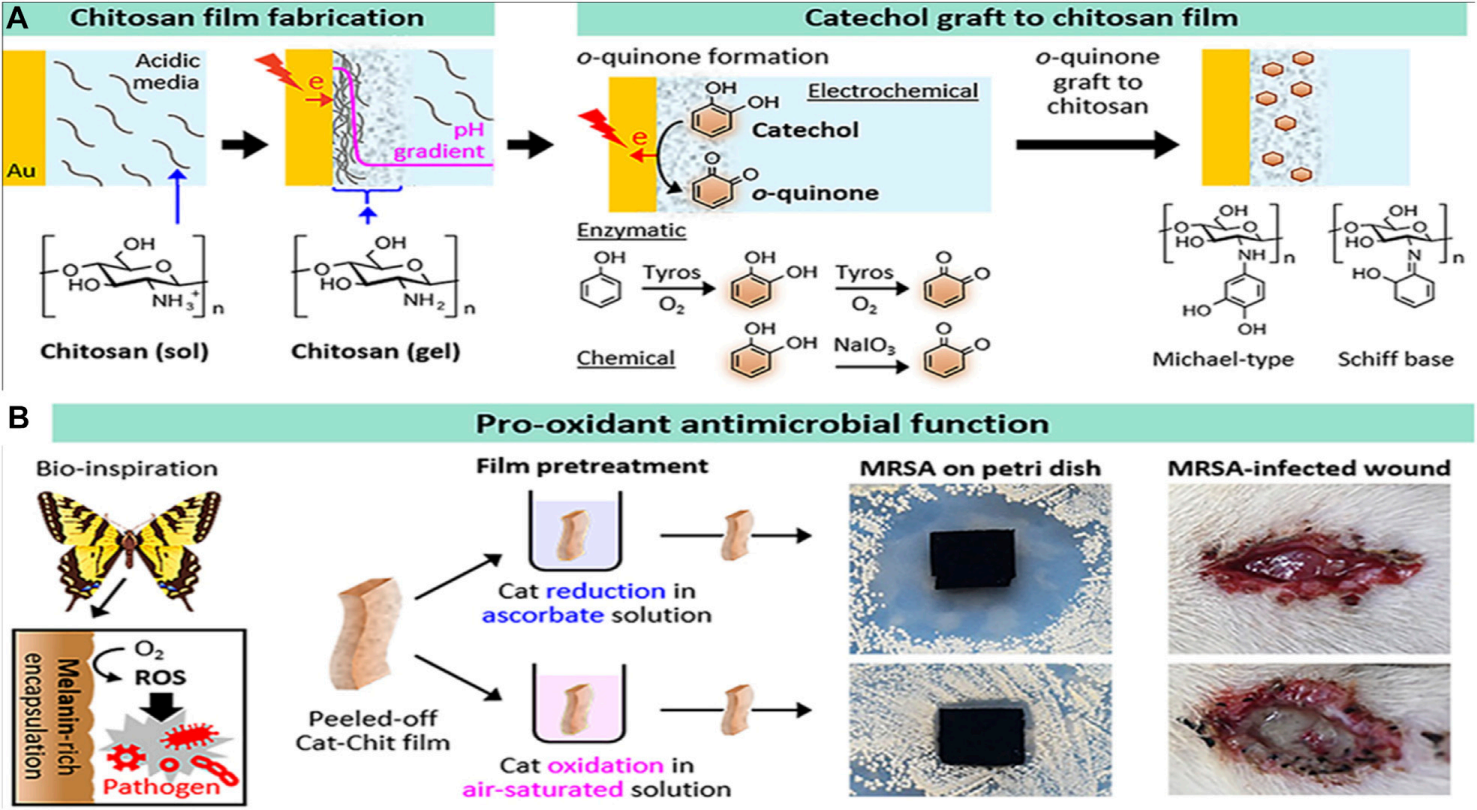
Catechol-grafted CH film with pro-antioxidant property. The fabrication process **(A)** and bioinspired pro-antioxidant activities associated with the generation of reactive oxygen species confer antimicrobial activities to wound healing. Adapted with modification from Kim et al. (2019) under CC BY version 4.0.

An unstable atom or molecule with an unpaired electron is known as a free radical. To a large extent, chronic diseases can be attributed to the free radical reaction (Pham-Huy et al., 2008). As free radicals are inherently unbalanced, they seek out and join forces with like-minded molecules and atoms in order to achieve equilibrium (Ngo et al., 2014). The formation of free radicals is inhibited by antioxidants by recycling or hastening their decay. The antioxidant activity in CH has recently been studied by numerous research groups (Wan et al., 2013). In healthy human cells, this unstable radical tends to bond with biological macromolecules, including proteins, lipids, and DNA, to become stable, damaging the DNA and proteins. Because of reduced cellular antioxidant defense mechanisms, radical-induced cell damage may propagate more rapidly (Lobo et al., 2010). Tan et al. (2022) used GRFT lipoic acid over CH via EDS/NHS coupling methods to improve the antioxidant activity. COS-caffeic acid conjugates with three different grafting degrees were synthesized by the N, N′-carbonyl diimidazole-mediated method to form an antioxidant film, which protects from UV damage (Liu et al., 2018a). Nowadays, CH-GRFT polymers are widely used in the food industry to prevent oxidation. According to a study, the phytoconstituents grafted on CH films show good antioxidant activity against corn oil storage via an ascorbate radicle-mediated reaction (Mittal et al., 2021). Catechol-GRFT-CH hydrogel-based films were fabricated by electrodepositing a film of amino-polysaccharides, and then oxidative GRFT catechol moieties to the film demonstrated excellent antioxidant, antimicrobial, and wound healing efficacy (Figure 9) (Kim et al., 2019).

### Anti-inflammatory activity of GRFT CH or COS

**FIGURE 10:**
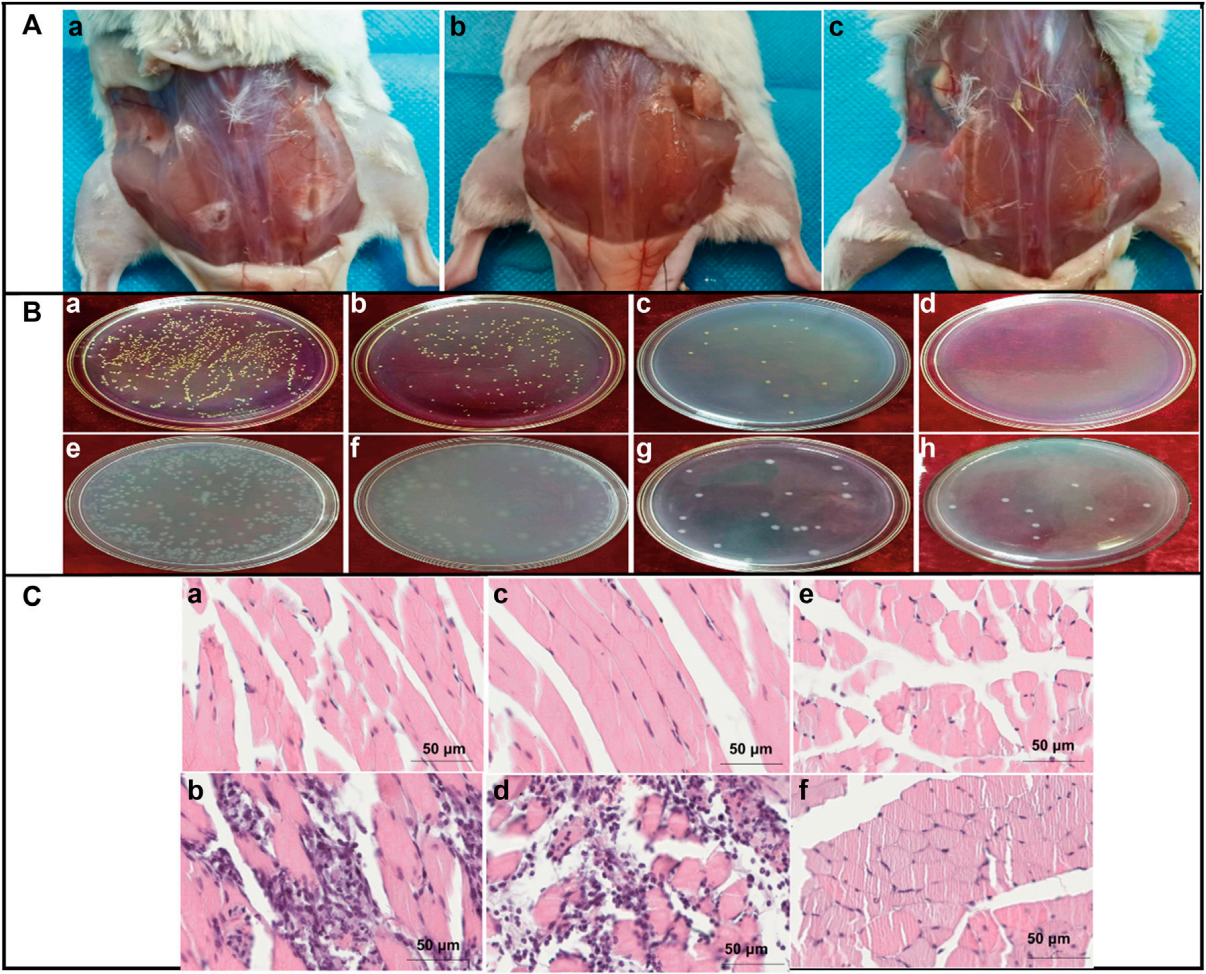
Antibacterial and anti-inflammatory activities of CH-copper complex coating catheters. **(A)** Wound situation after 5 days of subcutaneous implantations of (a) bare silicon, (b) pristine ch-copper, and (c) cu-modified ch-coated rubber catheters on the back of mice. **(B)** Morphologies of the bacterial colony of *E. coli* (a–d) and *S. aureus* (e–h) on the Petri dishes from low to high copper content as a result of the *in vitro* bactericidal activity value assay. **(C)** Paraffin section through H&E staining after implantation for 5 days; the implanted catheter induced no noticeable difference and inflammation on the left implanted sites or surrounding regions. Moreover, numerous inflammatory cells infiltrated the uncoated and CH-coated silicon rubbers. In addition, a few inflammatory cells were retrieved from the CH-copper-coated silicon rubber, demonstrating that the CH-copper-coated silicon rubber can significantly reduce the risk of device-associated bacterial colonization on the implant and then the risk of infection. Adapted with permission from Gu et al. (2022) under CC BY version 4.0.

**FIGURE 11:**
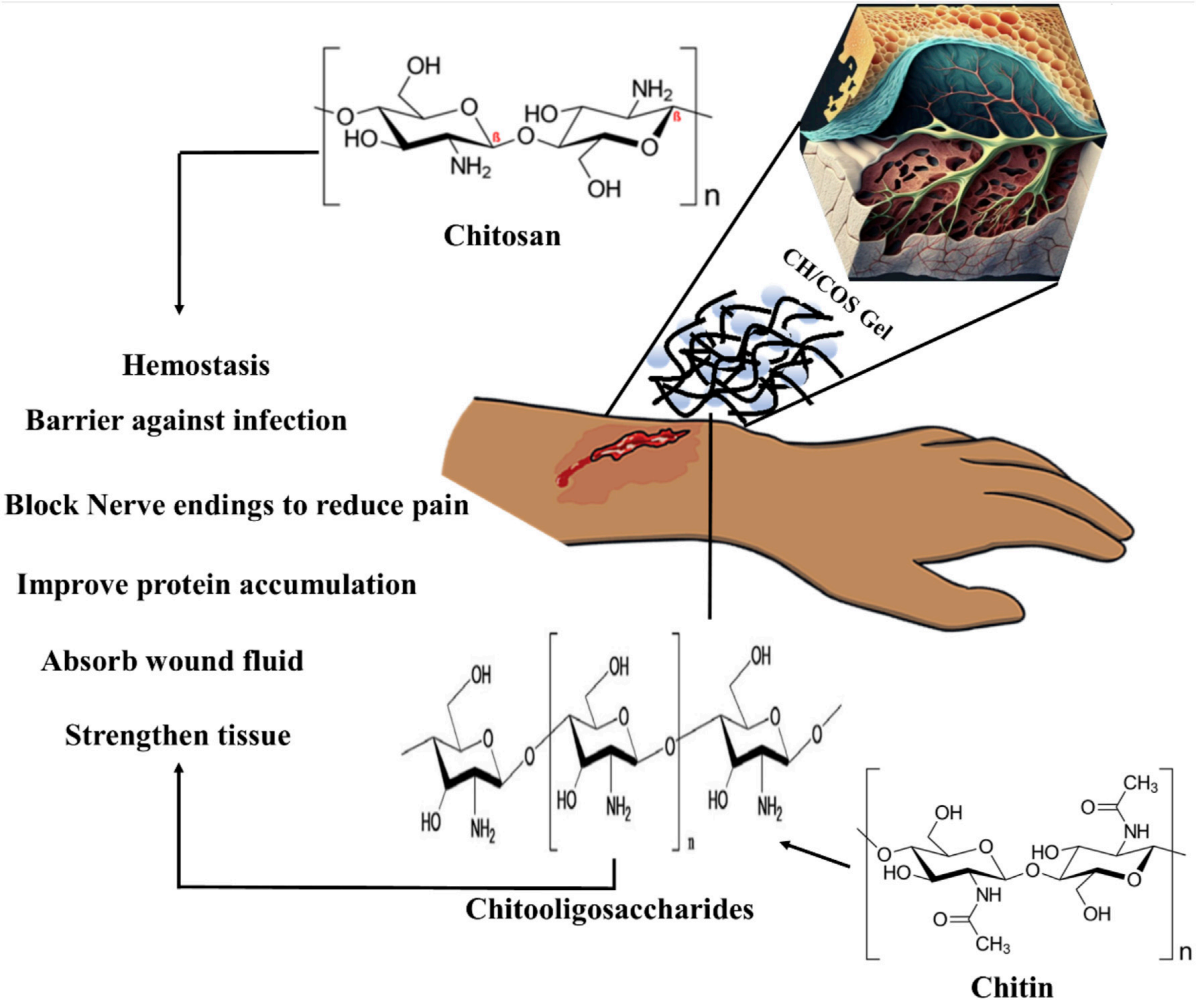
Mechanism of CH/COS-based hydrogels to promote wound healing. CH provides a non-protein matrix for three-dimensional tissue growth and activates macrophages for tumoricidal activity. Moreover, CH/COS stimulate cell proliferation and histoarchitectural tissue organization. In addition, CH is a hemostat that helps in natural blood clotting and blocks nerve endings, reducing pain.

Inflammation is an immune response that protects the body from pathogens. Inflammatory responses produce pro-inflammatory cytokines that help with tissue reparation or regeneration. If these responses become hyperactive, it leads to several metabolic disorders. CH has been suggested in multiple studies as a promising polysaccharide to deliver anticancer-based therapeutics (Adhikari and Yadav, 2018). Moreover, CH has antitumor properties, as it can stop tumors from arising, cause tumor cells to self-destruct, and stop tumors from spreading (Song et al., 2020). Several studies supported the anti-inflammatory effect of CH. The anti-inflammatory activity of copper- and CH-coated medical catheters was evaluated by *in vitro* and *in vivo* studies, showing promising results in the attenuation of inflammation responses (Figure 10) (Gu et al., 2022). Numerous researchers discovered dose-dependent attenuation of lipopolysaccharide (LPS) induced the secretion of TNF-α and IL-6 in the incubation medium in the presence of COS. Furthermore, the downregulation of the expression of cytokinin at the transcription level was observed. Furthermore, COS exposure also decreased the LPS-induced nitric oxide secretion in the medium (Mao et al., 2021a). Moreover, citronellol, a phytoconstituent grafted with COS, was synthesized to check the improved anti-inflammatory activity on the paw edema model. The COS-grafted citronellol suppressed the expression of TNF-α by promoting the secretion of IL-4 and IL-10, as indicated by *in vivo* results (Mao et al., 2021b). Carboxymethyl COS were investigated for the management of stenosis, one of the complications of Crohn’s disease (CD). The therapeutic efficacy of GRFT COS against stenosis was investigated and compared with the commercial drug 5-aminosalicylic acid over 28 days. The results indicated that GRFT COS significantly alleviated CD symptoms morphologically, hematologically, and pathologically, promoting the functional recovery of the intestinal epithelium in a dose-dependent manner. GFRT COS with carboxymethyl reduced infiltrations of inflammatory cells by regulating the IL-17A/PPAR-γ pathway and reduced fibro-proliferation and fibro-degeneration of the colon tissue by downregulating the TGF-β1/WT1 pathway, indicating promising candidature in the management of stenosis (Figure 11) (Hu, 2022 #2954).

### GRFT CH or COS ameliorate wound healing

**FIGURE 12:**
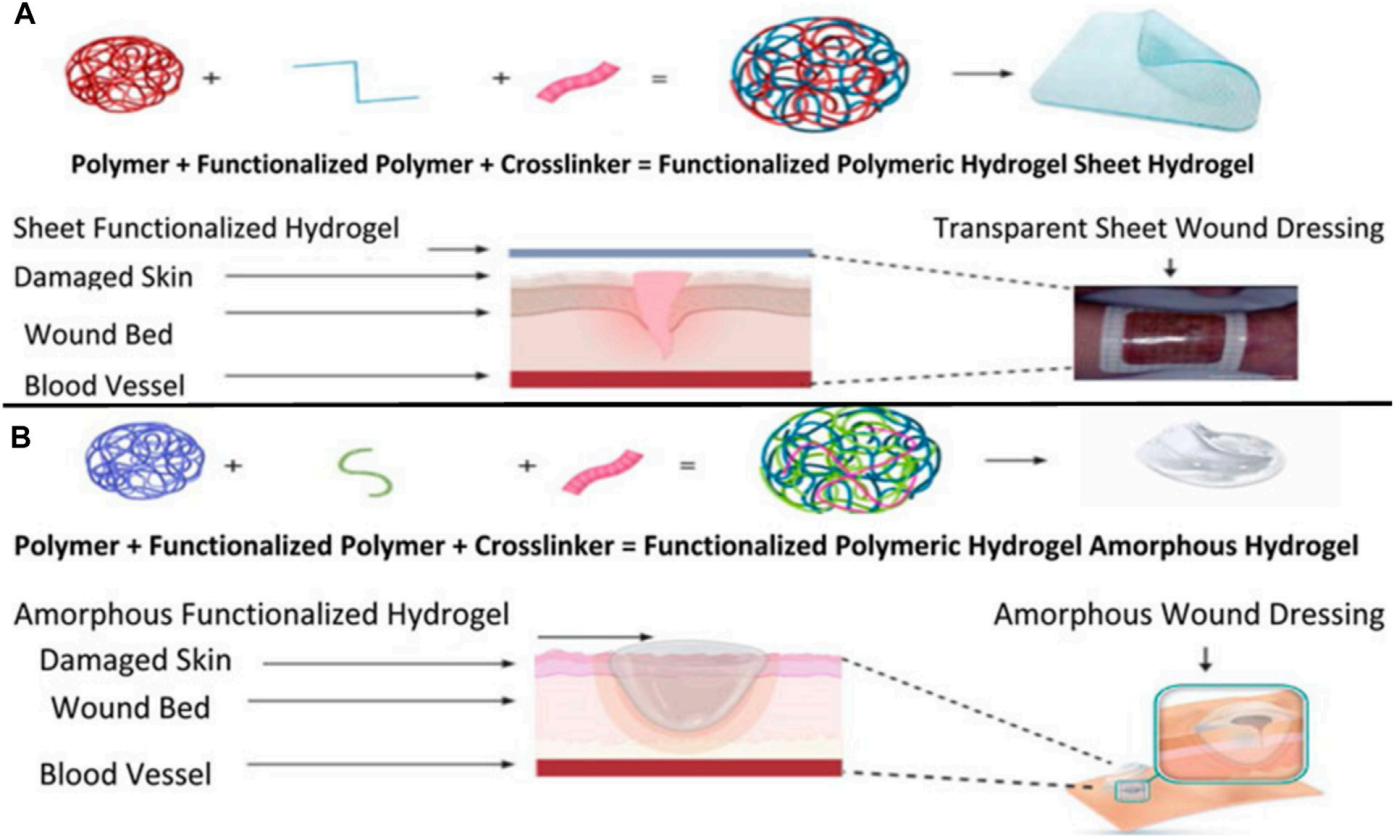
Synthesis and application of sheet-functionalized CH hydrogels for wound healing purposes **(A)**. Synthesis and application of a functionalized amorphous GRFT polymer hydrogel **(B)**. Adapted with permission from Kibungu et al. (2021) under CC BY version 4.0.

Human skin is the largest organ that accounts for 10% of the total body weight and is a barrier to the environment. Along with serving as a physical obstruction, the skin also serves as a sensory detector, thermoregulator, fluid homeostat, and immune regulator. Usually, a complicated and dynamic process allows the human body to repair damaged skin with a little scar. Coagulation and hemostasis, inflammation, proliferation, and remodeling are the four time-dependent phases that make up the various processes of acute tissue repair. Scars on the skin are responsible for wound healing, and their elimination is a significant challenge (Figure 11) (Liu et al., 2018b). This challenge can be overcome by using injectable hydrogel-based grafted hydroxypropyl CH and microcrystalline cellulose for wound healing (Huang et al., 2022). Additionally, in a recent review, a multifunctional CH hyaluronic acid three-dimensional hydrogel indicated that the network has high water absorption property that can be significantly applied in the management of inflammatory bowel diseases with emphasis on adhesion, synergistic therapy, pH sensitivity, particle size, and temperature sensitivity (Ouyang et al., 2023). Hydrogel dressings can exist in two main forms: amorphous and sheet dressings (Kibungu et al., 2021). Amorphous hydrogel dressings exhibit low extrudate absorptive capacity due to their high water contents, while sheet dressings are mainly applied during the final phase and phlebitis. Furthermore, clear and transparent sheet hydrogels can be used to monitor wound healing progress in real-time (Figure 12). Moreover, Shuang et al. demonstrated that injectable hydrogels significantly improved the wound healing process by preventing scar formation (Liu et al., 2022). Chronic diabetes mellitus is characterized by prolonged hyperglycemia, and this environment promotes the formation of biofilms, which makes wounds challenging to treat (Burgess et al., 2021). Several investigations reported regarding the grafted CH in the amelioration of diabetic wound healing activity (Burgess et al., 2021). In addition, a carboxymethyl CH and carboxymethyl cellulose-based hydrogel shows dual drug delivery with acceleration in diabetic wound healing (Chang et al., 2022). An *in situ* cross-linked carboxymethyl CH/oxidized dextran/poly-y-glutamic acid/hydrogel exhibited significant inhibition of wound pathogenic bacterial growth and promoted wound healing (Chen et al., 2023).

### Therapeutic activity of bioactive grafted CH

The biodegradability and usefulness of CH in several therapeutic contexts led to its incorporation into a range of drug delivery systems, some of which are described here. Current nanomaterials, with their unique properties and potential for use in medicine, are proving to be rather useful. CH as a carrier combines various metallic, polymeric, and lipophilic nanoparticles for anti-inflammatory, wound-healing, and antibacterial purposes (Augustine et al., 2020). For oral insulin delivery, insulin-loaded carboxylated CH-GFRT polymethyl acrylate nanoparticles are reported (Cui et al., 2009). Electrospun CH has been the subject of numerous previous research efforts due to their potential uses in skin regeneration. Electrospun core-shelled fibers are now being developed for enhanced therapeutic action. Core CH/PLGA fibers were fabricated by Lili et al. by electrospinning, which demonstrated improved cytocompatibility, cell viability, and cell adhesion to promote cell migration through the membranes. It is possible that the membranes obtained could be used in tissue engineering or as a part of a dressing for a wound (Frigaard et al., 2022). In another study, vancomycin-loaded GFRT-CH CL using glutaraldehyde demonstrated polyampholyte behavior and displayed charge-reversible behavior after treatment with sodium chloride and a drug loading efficacy of 91.3%. This polyaniline pH-responsive carrier microgels exhibited significant lysozyme-receptiveness and efficiency in the management of inflammatory bowel diseases (Li et al., 2023). Cytotoxicity reports on CH/COS on different cells are given in Table 3.

**TABLE 3:** Cytotoxicity of chitosan and chito-oligosaccharides on different cells.

Nature of CH	Carrier system	Active ingredient	Cell line	Result	Reference
CH (76%)	CH-NPs	Doxorubicin	HT-1197 cells	Unloaded NPs showed no significant decline in cell viability at tested concentrations	Ali et al. (2020)
CH (95%)	CH-NPs	Myricetin	Caco-2 cells	Myricetin-loaded NPs showed >90% cell viability	Sang et al. (2020)
CH	CH nanospheres	Curcumin	Kerman male breast cancer cells	Cytotoxicity investigation indicated a dose-dependent reaction against cancer breast cells after 24 h incubation	Afzali et al. (2021)
COS	Stearic acid-grafted COS-NPs	Doxorubicin	A549, LLC, and SKOV3 cells	Cytotoxicities of shell-crosslinked COS-SA/DOX were highly enhanced in all cell lines compared to those of unmodified COS	Hu et al. (2008)
COS	Glycosylated COS-NPs	-	HepG2	*In vitro* analysis using HepG2 cells suggested that the NPs have a more specific uptake capacity	Manivasagan et al. (2016)

### Modification and drug applications of CH and COS

GRF synthetic polymers onto a natural polymer are a practical way to modify the latter’s properties with minimal impact on the former. Natural polysaccharides may be a promising starting material for graft copolymer synthesis due to their structural variety and solubility in water. The biopolymer backbone is often grafted onto a vinyl or acryl monomer by polymerization to create a copolymer. GRF non-vinyl/acryl monomers are chemically very different from grafting vinyl/acryl monomers. By polycondensation, GRF copolymerization with monomers other than vinyl chloride and acrylate can be achieved. Unfortunately, the polysaccharide backbone is highly susceptible to the high temperatures and severe conditions of the conventional polycondensation reactions; hence, this method has not been frequently used for making graft copolymers of polysaccharides (Kalia et al., 2013). Of the many purposes, the primary purpose of surface modification is improving a surface polymer’s wettability, biocompatibility, mechanical properties, etc. Two significant types of grafting may be considered, i.e., GRF with a single monomer and GRF with a mixture of two (or more) monomers (Sakhare and Rajput, 2017). The endo-chitosanase enzyme degrades CH to COS, a functional food. A newly isolated *Mitsuaria* sp. C4 produced a 34-kDa COS with a 38.46% recovery rate and 4.79-fold purification (Chen et al., 2021).

CH-GRF using single vinyl monomers for drug delivery and other applications has been reported by Subramanian and Vijayakumar (2012). A study was conducted on CH-GRF using the hydrophilic itaconic acid and the comparatively more hydrophobic 2-hydroxyethyl methacrylate as comonomers, and the resultant CH-GRF was evaluated as a tunable drug carrier with tramadol hydrochloride. Hydrophilic medicines and sensitive proteins may be transported as CH-GRF-PLA particles (Hawthorne et al., 2022). CH microspheres containing polyacrylamide grafts were crosslinked with glutaraldehyde to encapsulate indomethacin, demonstrating regulated release via molecular diffusion phenomena (Kumbar and Aminabhavi, 2003).

Poly(ethylene glycol), poly(2-hydroxyethyl acrylate), poly(2-ethyl-2-oxazoline), and poly(2-hydroxyethyl) GRFT onto CH chemically modified (N-vinyl pyrrolidone) and further processed to form NPs. These NPs were tested on sheep mucosa, and the results showed improvement for modified chitosan nanoparticles diffused compared to unmodified chitosan nanoparticles (N-vinyl pyrrolidone) (Ways et al., 2022). In contrast, a different study used a CH-GRF-poly(N-vinyl caprolactam) copolymer to coat gold nanoparticles that had been modified with a thioglycolic acid ligand. The effectiveness of the produced nanocarriers in treating MCF-7 breast cancer cells *in vitro* was studied. The release of cisplastin from nanoparticles was found most accurately, compared with cisplastin alone (Banihashem et al., 2020).

**FIGURE 13:**
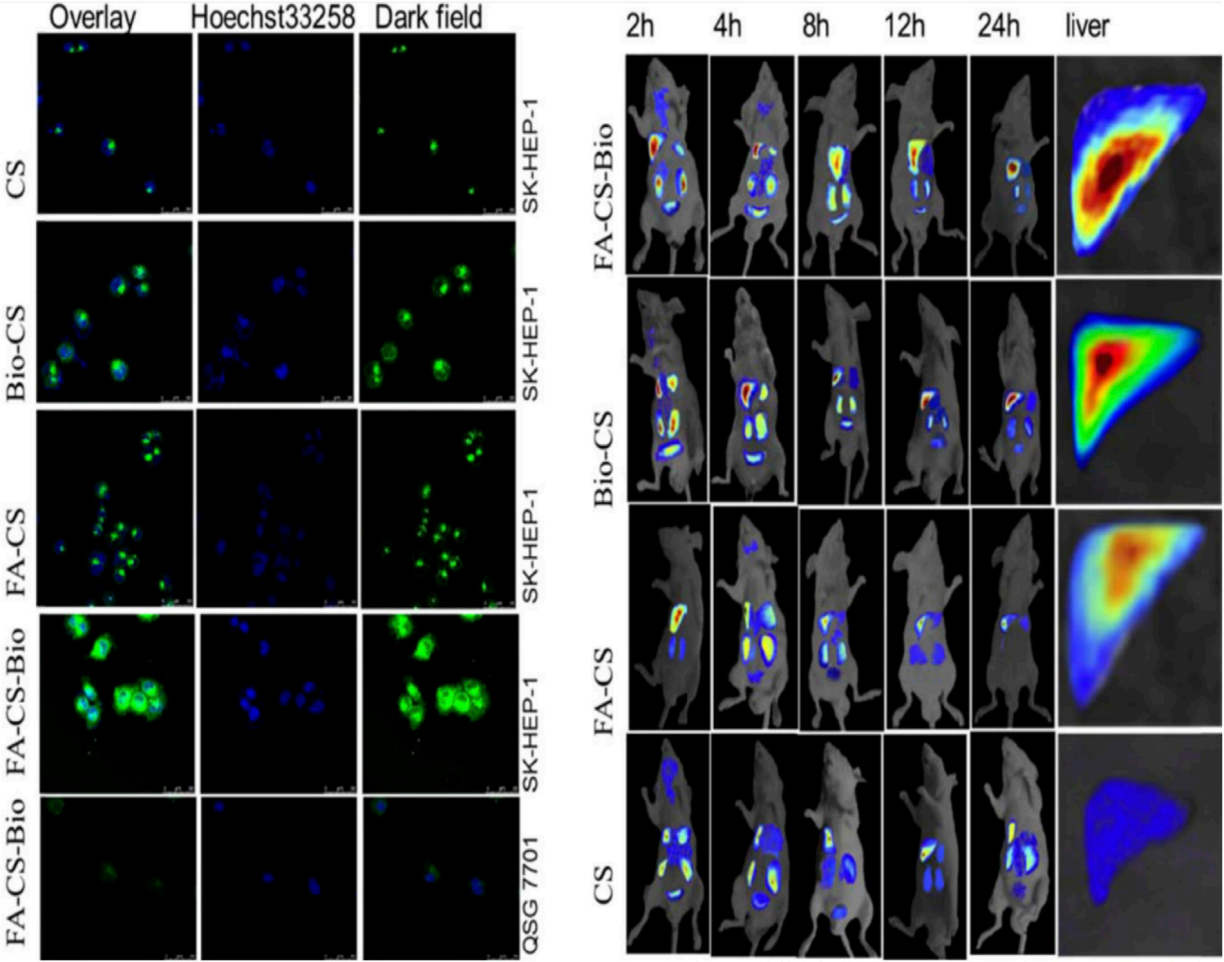
Confocal detection of endocytosis of liver cancer cells by different nanomaterials. *In vivo* imaging of different nanomaterials in a mouse orthotopic liver cancer model. Adapted with permission from Cheng and Dai (2022) under CC BY version 4.0.

**FIGURE 14:**
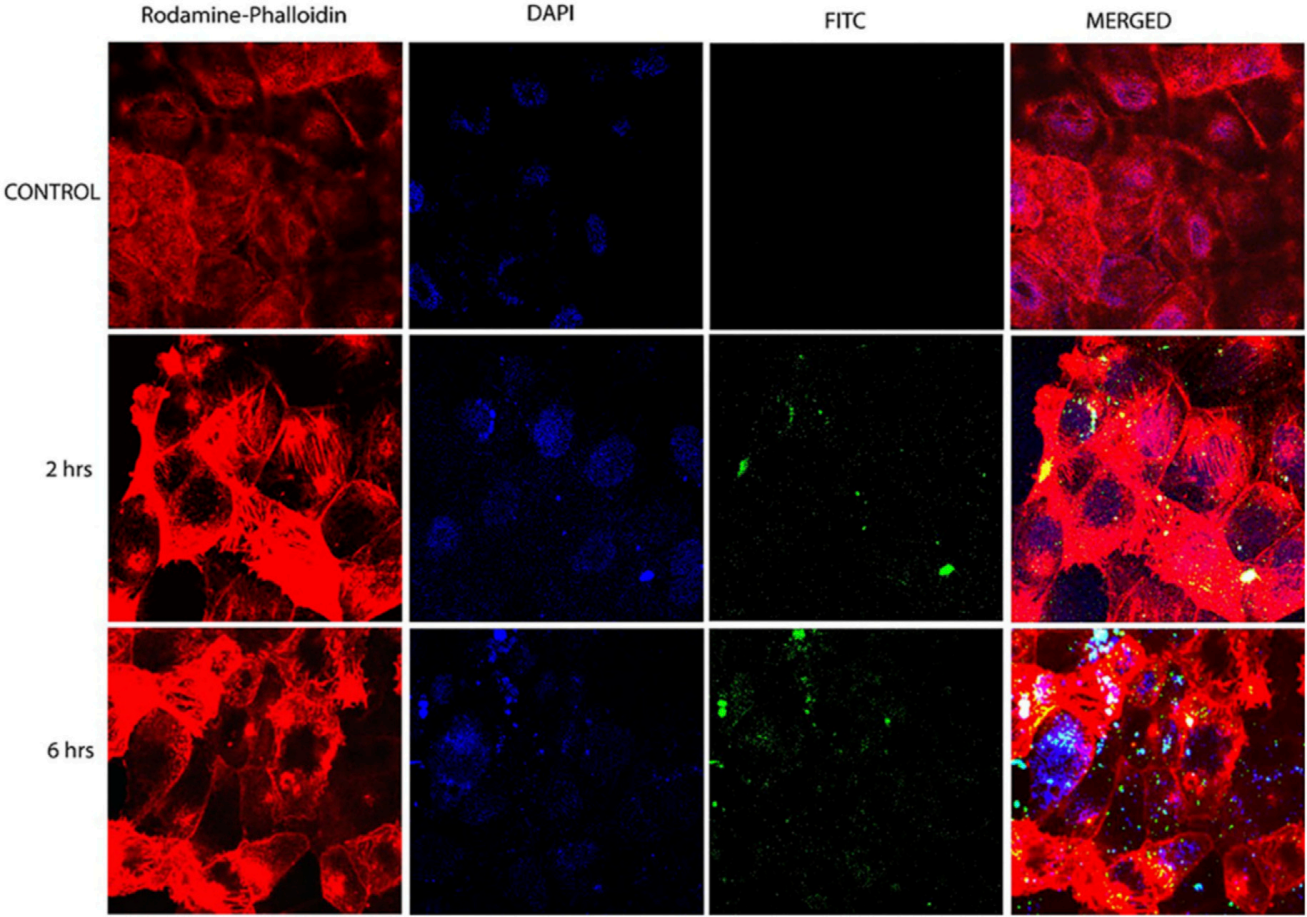
Confocal microscopy images of CaCo-2 cells after 6 h incubation at 37°C with FITC-tagged Cs-SLN of either amphotericin b or paromomycin. Adapted with permission from Parvez et al. (2020) under CC BY version 4.0.

**FIGURE 15:**
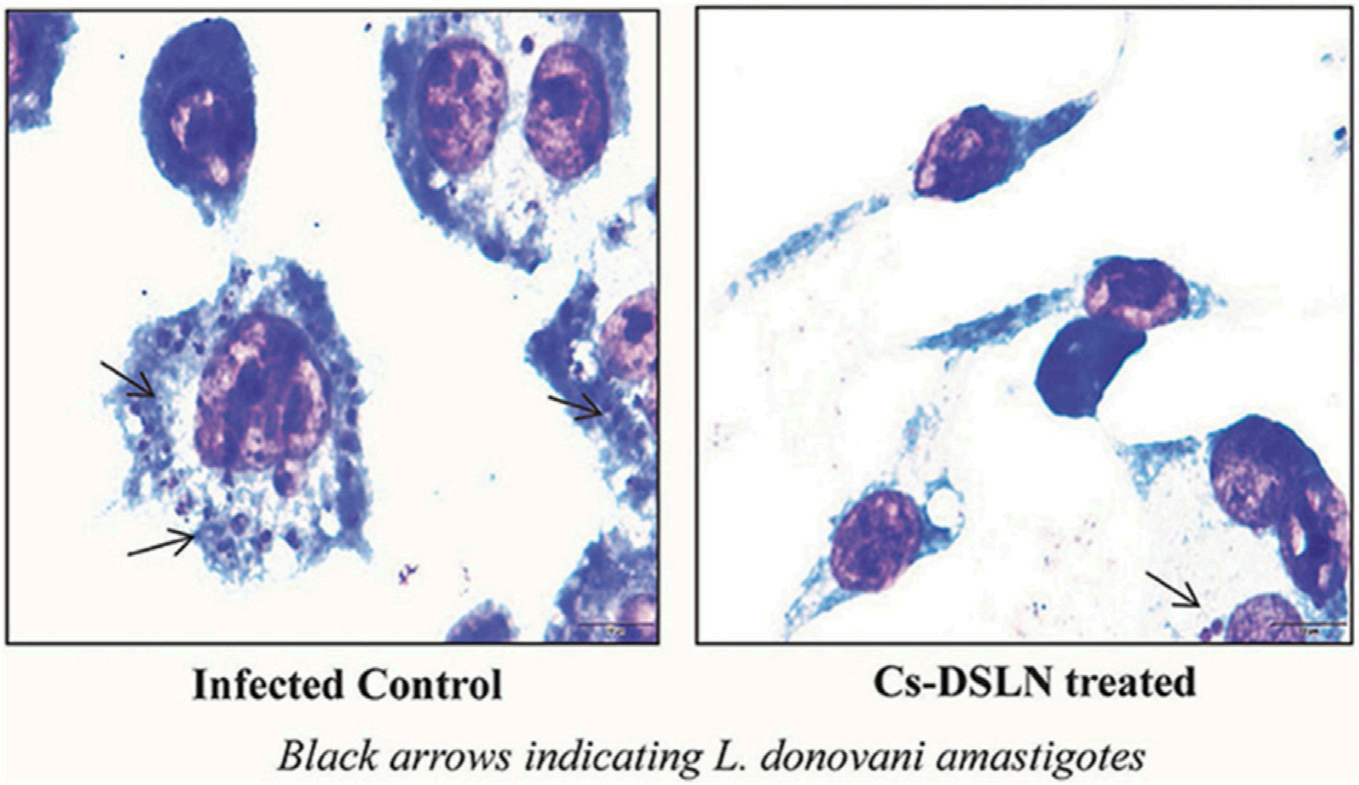
Micrographs of Cs-SLN-treated and untreated *L. donovani*-injected J774A1 macrophages. Adapted with permission from Parvez et al. (2020) under CC BY version 4.0.

CH-based injectable hydrogel gelation can be triggered by physical stimuli or chemical reactions, making them less invasive. For the purposes of drug delivery, tissue engineering, and cancer treatment, many injectable hydrogels based on CH have been explored and their chemical compositions optimized (Nawrotek et al., 2016). Injectable hydrogels based on the CH-g-(mPEG-b-PCL) graft copolymer showed temperature- and pH-responsive sol-gel phase transitions with an effective release of drugs for up to 2 weeks (Jommanee et al., 2018). In another study, CH-based implants employed in CH can form complex coacervates and insoluble ionic complexes with a range of water-soluble anionic polymers, depending on the charge density, with potential applications in tissue regeneration and cell transplantation (Sharma et al., 2021). CH-NPs formulated with carbopol and CH-coated niosomal timolol maleate have been shown to be effective in achieving sustained release, as have GRFT-CH with poly(N-isopropyl acrylamide) to create a thermally responsive ocular delivery vehicle. Gupta et al. (2014) reported microwave-induced graft copolymer synthesis on delignified *Grewia optiva* fiber for dye removal. Adsorption of harmful methylene dye from aqueous systems has been investigated using grafted polymers. Targeting of different GRFT-CH with 5-fluorouracil nano-formulations on hepatoma cells in different tissues of a mouse orthotopic liver cancer transplantation model showed the fluorescence intensity of the endocytosed nanoparticles (Figure 13A) (Cheng and Dai, 2022). Moreover, the results showed a higher green fluorescence signal than unmodified CH conjugated 5-fluorouracil. In order to further confirm this, an *in vivo* targeting investigation was performed. The results indicated that treatment with modified CH conjugated with drugs demonstrated the strongest photon number in liver cancer tissues, compared with unmodified CH conjugation (Figure 13B) (Cheng and Dai, 2022). Amphotericin B and paromomycin fortified with GRFT-CH nanocarriers via oral routes for enhanced anti-leishmanial activity demonstrated increase drug internalization into CaCo-2 cells via FITC-tagged Cs-SLN in a time-dependent way. In addition, no toxic response was observed, as confirmed by no change in the morphology of cells (Figure 14) (Parvez et al., 2020). Additionally, anti-leishmanial activity of conjugated GRFT-CH-amphotericin B was tested against *L. donovani*-infected macrophages. At the tested concentration (1 μg/mL), the intra-cellular amastigotes were significantly diminished, compared with free amphotericin B (Figure 15) (Parvez et al., 2020).

### Green synthesis of metallic nanoparticles using grafted CH and COS

Metal NPs could be synthesized using a wide range of chemical and physical processes (Dikshit et al., 2021). The use of poisonous solvents, the creation of dangerous by-products, and the need for significant energy are just a few of the issues with these processes. Therefore, it is crucial to create eco-friendly methods of producing metallic nanoparticles (MNPs). Using the many biological resources already present in the natural world is a promising method for achieving this objective.

**FIGURE 16:**
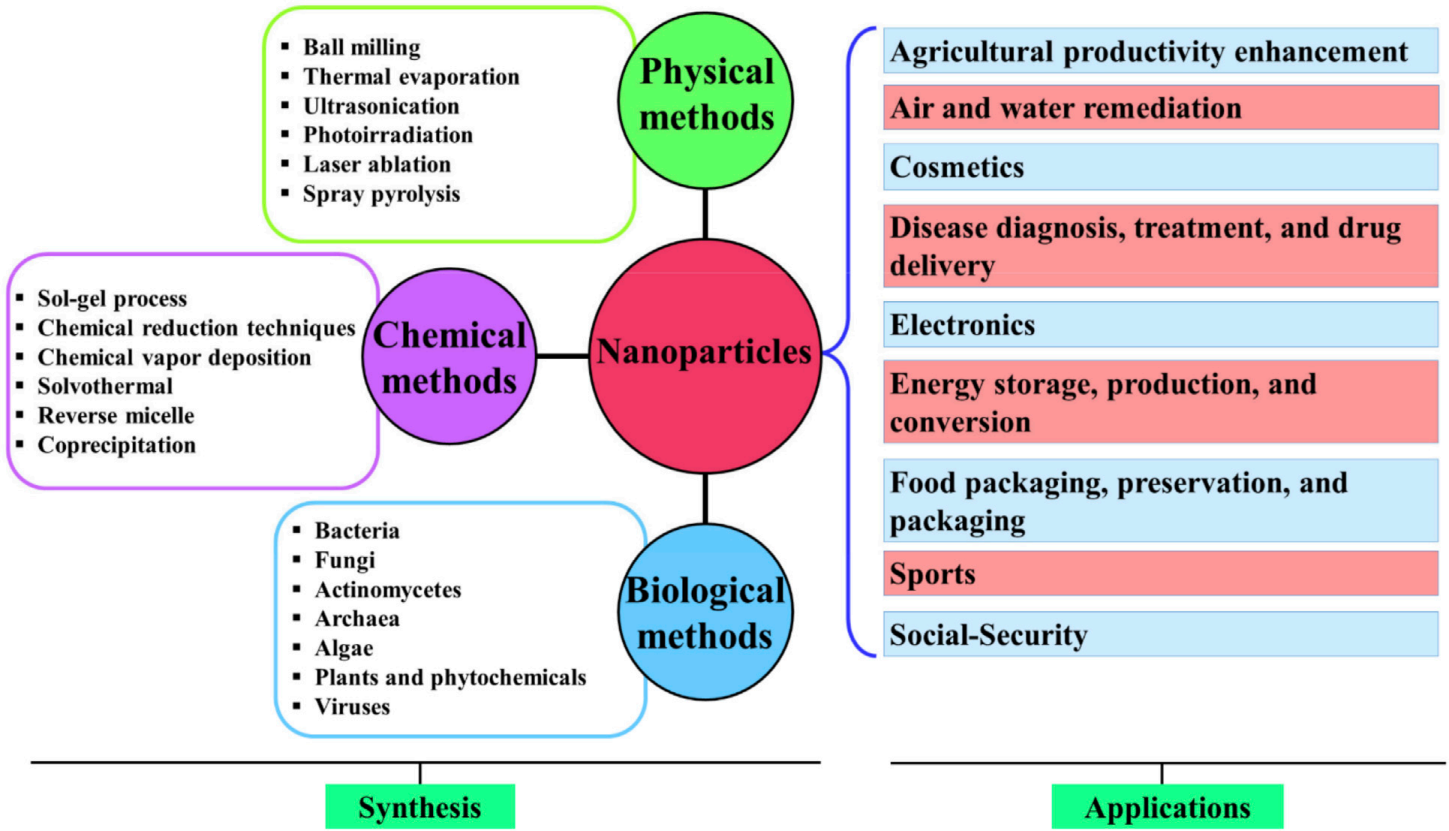
Schematic representation of various methods adopted for the synthesis of NPs and their applications. Adapted with permission from Dikshit et al. (2021) under CC BY version 4.0.

A schematic representation of various methods adopted for NP synthesis and their applications is given in Figure 16.

An effect of CH on the synthesis and functionalization of MNPs was studied by several researchers (Phan et al., 2021). The synthesis and functionalization of MNPs can be influenced by corrosive stress. An electrostatic interaction occurs between the positively charged cationic polymer CH and the negatively charged NPs (which have a negative capping agent), or an interaction occurs via the absorption of CH on the metal nanoparticle surface, resulting in a CH shell around the NPs.

## Conclusion

CH is the most promising polysaccharide for various applications because of its cationic nature, which is not shared by other polysaccharides. Several strategies for altering the surface characteristics of CH have emerged in recent years. Biomedical and environmental applications of CH and COS materials have demonstrated remarkable physicochemical, mechanical, and biological properties. Numerous studies reporting success with applications where CH/COS is the primary component testify to the commercial significance of this natural polymer. GRF copolymerization techniques have emerged as a means of improving upon the already outstanding properties of CH. These approaches promise efficient ways to engineer CH to incorporate the properties and functionalities of interest. Although CH is superior to other polysaccharides in several aspects, it is essential to understand how modifying its surface with various functional groups might enhance its ultimate applications. To further develop and expand the multifunctional uses of CH, it is essential to expand our understanding of the structure and chemistry of diverse polymerization activities. Drug delivery is just one of the many potential uses for polysaccharide grafted/crosslinked copolymers, which are shown to be both practical and unique in the future of CH/COS.

## Abbreviations

COS, chito-oligosaccharides; CH, chitosan; CLSM, confocal microscopy images; CL, cross-linking; DNA, deoxyribonucleic acid; DSC, differential scanning calorimetry; EG, enzymatic grafting; FA-CS, folic acid-grafted chitosan; FTIR, Fourier infrared spectroscopy; FRP, free radical polymerization; GRFT, grafted; GRF, grafting; LP, living polymerization; MNPs, metallic nanoparticles; NPs, nanoparticles; PG, photochemical grafting; PLGA, poly(lactide-co-glycolide); PEG, polyethylene glycol; PVA, polyvinyl alcohol; RG, radiation grafting; ROP, ring-opening polymerization; SEM, scanning electron microscopy; XRD, X-ray diffraction.

## References

[B1] Abd El-Hack M. E. El-Saadony M. T. Shafi M. E. Zabermawi N. M. Arif M. Batiha G. E. (2020). Antimicrobial and antioxidant properties of chitosan and its derivatives and their applications: A review. Int. J. Biol. Macromol. 164, 2726–2744. 10.1016/j.ijbiomac.2020.08.153 32841671

[B2] Adhikari H. S. Yadav P. N. (2018). Anticancer activity of chitosan, chitosan derivatives, and their mechanism of action. Int. J. Biomaterials 2018, 1–29. 10.1155/2018/2952085 PMC6332982 30693034

[B3] Afzali E. Eslaminejad T. Yazdi Rouholamini S. E. Shahrokhi-Farjah M. Ansari M. (2021). Cytotoxicity effects of curcumin loaded on chitosan alginate nanospheres on the kmbc-10 spheroids cell line. Int. J. Nanomedicine 16, 579–589. 10.2147/ijn.s251056 33531802 PMC7846832

[B4] Ahmad Z. Salman S. Khan S. A. Amin A. Rahman Z. U. Al-Ghamdi Y. O. (2022). Versatility of hydrogels: From synthetic strategies, classification, and properties to biomedical applications. Gels 8 (3), 167. 10.3390/gels8030167 35323280 PMC8950628

[B5] Ali M. S. Metwally A. A. Fahmy R. H. Osman R. (2020). Chitosan-coated nanodiamonds: Mucoadhesive platform for intravesical delivery of doxorubicin. Carbohydr. Polym. 245, 116528. 10.1016/j.carbpol.2020.116528 32718632

[B6] Augustine R. Rehman S. R. U. Ahmed R. Zahid A. A. Sharifi M. Falahati M. (2020). Electrospun chitosan membranes containing bioactive and therapeutic agents for enhanced wound healing. Int. J. Biol. Macromol. 156, 153–170. 10.1016/j.ijbiomac.2020.03.207 32229203

[B7] Bahramzadeh E. Yilmaz E. Adali T. (2019). Chitosan-graft-poly(N-hydroxy ethyl acrylamide) copolymers: Synthesis, characterization and preliminary blood compatibility *in vitro* . Int. J. Biol. Macromol. 123, 1257–1266. 10.1016/j.ijbiomac.2018.12.023 30521908

[B8] Bakshi P. S. Selvakumar D. Kadirvelu K. Kumar N. (2020). Chitosan as an environment friendly biomaterial–a review on recent modifications and applications. Int. J. Biol. Macromol. 150, 1072–1083. 10.1016/j.ijbiomac.2019.10.113 31739057

[B9] Banihashem S. Nezhati M. N. Panahi H. A. (2020). Synthesis of chitosan-grafted-poly(N-vinylcaprolactam) coated on the thiolated gold nanoparticles surface for controlled release of cisplatin. Carbohydr. Polym. 227, 115333. 10.1016/j.carbpol.2019.115333 31590864

[B10] Bourgat Y. Tiersch B. Koetz J. Menzel H. (2021). Enzyme degradable polymersomes from chitosan-g-[poly-l-lysine-block-ε-caprolactone] copolymer. Macromol. Biosci. 21 (1), 2000259. 10.1002/mabi.202000259 33289254

[B11] Bravo A. Likitvivatanavong S. Gill S. S. Soberón M. (2011). Bacillus thuringiensis: A story of a successful bioinsecticide. Insect Biochem. Mol. Biol. 41 (7), 423–431. 10.1016/j.ibmb.2011.02.006 21376122 PMC3689885

[B12] Brocas A.-L. Mantzaridis C. Tunc D. Carlotti S. (2013). Polyether synthesis: From activated or metal-free anionic ring-opening polymerization of epoxides to functionalization. Prog. Polym. Sci. 38 (6), 845–873. 10.1016/j.progpolymsci.2012.09.007

[B13] Bundschuh M. Filser J. Lüderwald S. McKee M. S. Metreveli G. Schaumann G. E. (2018). Nanoparticles in the environment: Where do we come from, where do we go to? Environ. Sci. Eur. 30 (1), 6–17. 10.1186/s12302-018-0132-6 29456907 PMC5803285

[B14] Burgess J. L. Wyant W. A. Abdo Abujamra B. Kirsner R. S. Jozic I. (2021). Diabetic wound-healing science. Med. Kaunas. 57 (10), 1072. 10.3390/medicina57101072 PMC8539411 34684109

[B15] Cai D. Han C. Liu C. Ma X. Qian J. Zhou J. (2020). Chitosan-capped enzyme-responsive hollow mesoporous silica nanoplatforms for colon-specific drug delivery. Nanoscale Res. Lett. 15 (1), 123. 10.1186/s11671-020-03351-8 32488526 PMC7266918

[B16] Camarillo R. C. Pérez O. S. Avelizapa N. R. Ramírez M. G. Avelizapa L. R. (2004). Chitosanase activity in *Bacillus thuringiensis* . Folia Microbiol. 49 (1), 94–96. 10.1007/bf02931653 15114873

[B17] Campos E. V. R. Oliveira J. L. Fraceto L. F. (2017). Poly(ethylene glycol) and cyclodextrin-grafted chitosan: From methodologies to preparation and potential biotechnological applications. Front. Chem. 5, 93. 10.3389/fchem.2017.00093 29164107 PMC5681902

[B18] Cao W. Yan J. Liu C. Zhang J. Wang H. Gao X. (2020). Preparation and characterization of catechol-grafted chitosan/gelatin/modified chitosan-AgNP blend films. Carbohydr. Polym. 247, 116643. 10.1016/j.carbpol.2020.116643 32829790

[B19] Chang G. Dang Q. Liu C. Wang X. Song H. Gao H. (2022). Carboxymethyl chitosan and carboxymethyl cellulose based self-healing hydrogel for accelerating diabetic wound healing. Carbohydr. Polym. 292, 119687. 10.1016/j.carbpol.2022.119687 35725178

[B20] Chapelle C. David G. Caillol S. Negrell C. Desroches Le Foll M. (2021). Advances in chitooligosaccharides chemical modifications. Biopolymers 112 (9), e23461. 10.1002/bip.23461 34115397

[B21] Charoenpol A. Crespy D. Schulte A. Suginta W. (2023). Marine chitin upcycling with immobilized chitinolytic enzymes: Current state and prospects. Green Chem. 25, 467–489. 10.1039/d2gc02013k

[B22] Chen D. Chen C. Zheng X. Chen J. He W. Lin C. (2021). Chitosan oligosaccharide production potential of mitsuaria sp. c4 and its whole-genome sequencing. Front. Microbiol. 12, 695571. 10.3389/fmicb.2021.695571 34421850 PMC8374441

[B23] Chen Z. Yao J. Zhao J. Wang S. (2023). Injectable wound dressing based on carboxymethyl chitosan triple-network hydrogel for effective wound antibacterial and hemostasis. Int. J. Biol. Macromol. 225, 1235–1245. 10.1016/j.ijbiomac.2022.11.184 36435472

[B24] Cheng M. Dai D. (2022). Inhibitory of active dual cancer targeting 5-Fluorouracil nanoparticles on liver cancer *in vitro* and *in vivo* . Front. Oncol. 12, 971475. 10.3389/fonc.2022.971475 35992879 PMC9389539

[B25] Choudhary S. Sharma K. Sharma V. Kumar V. (2020). Grafting polymers. Reactive and functional polymers volume two: Modification reactions, compatibility and blends, 199–243.

[B26] Chung T.-W. Lu Y.-F. Wang S.-S. Lin Y.-S. Chu S.-H. (2002). Growth of human endothelial cells on photochemically grafted Gly–Arg–Gly–Asp (GRGD) chitosans. Biomaterials 23 (24), 4803–4809. 10.1016/s0142-9612(02)00231-4 12361619

[B27] Cui F. Qian F. Zhao Z. Yin L. Tang C. Yin C. (2009). Preparation, characterization, and oral delivery of insulin loaded carboxylated chitosan grafted poly(methyl methacrylate) nanoparticles. Biomacromolecules 10 (5), 1253–1258. 10.1021/bm900035u 19292439

[B28] Dara P. K. Mahadevan R. Digita P. A. Visnuvinayagam S. Kumar L. R. G. Mathew S. (2020). Synthesis and biochemical characterization of silver nanoparticles grafted chitosan (chi-Ag-NPs): *In vitro* studies on antioxidant and antibacterial applications. SN Appl. Sci. 2 (4), 665. 10.1007/s42452-020-2261-y

[B29] de Sousa Victor R. Marcelo da Cunha Santos A. Viana de Sousa B. de Araújo Neves G. Navarro de Lima Santana L. Rodrigues Menezes R. (2020). A review on chitosan’s uses as biomaterial: Tissue engineering, drug delivery systems and cancer treatment. Materials 13 (21), 4995. 10.3390/ma13214995 33171898 PMC7664280

[B30] Dey A. Haldar U. De P. (2021). Block copolymer synthesis by the combination of living cationic polymerization and other polymerization methods. Front. Chem. 9, 644547. 10.3389/fchem.2021.644547 34262892 PMC8273170

[B31] Diao Y. Yu X. Zhang C. Jing Y. (2020). Quercetin-grafted chitosan prepared by free radical grafting: Characterization and evaluation of antioxidant and antibacterial properties. J. Food Sci. Technol. 57 (6), 2259–2268. 10.1007/s13197-020-04263-2 32431352 PMC7230106

[B32] Dikshit P. K. Kumar J. Das A. K. Sadhu S. Sharma S. Singh S. (2021). Green synthesis of metallic nanoparticles: Applications and limitations. Catalysts 11 (8), 902. 10.3390/catal11080902

[B33] Ding W.-Y. Zheng S.-D. Qin Y. Yu F. Bai J.-W. Cui W.-Q. (2019). Chitosan grafted with β-cyclodextrin: Synthesis, characterization, antimicrobial activity, and role as absorbefacient and solubilizer. Front. Chem. 6, 657. 10.3389/fchem.2018.00657 30687698 PMC6335354

[B34] Elieh-Ali-Komi D. Hamblin M. R. (2016). Chitin and Chitosan: Production and application of versatile biomedical nanomaterials. Int. J. Adv. Res. 4 (3), 411–427. PMC5094803 27819009

[B35] Eze F. N. Jayeoye T. J. Singh S. (2022). Fabrication of intelligent pH-sensing films with antioxidant potential for monitoring shrimp freshness via the fortification of chitosan matrix with broken Riceberry phenolic extract. Food Chem. 366, 130574. 10.1016/j.foodchem.2021.130574 34303209

[B36] Frigaard J. Jensen J. L. Galtung H. K. Hiorth M. (2022). The potential of chitosan in nanomedicine: An overview of the cytotoxicity of chitosan based nanoparticles. Front. Pharmacol. 13, 880377. 10.3389/fphar.2022.880377 35600854 PMC9115560

[B37] Fuster M. G. Montalbán M. G. Carissimi G. Lima B. Feresin G. E. Cano M. (2020). Antibacterial effect of chitosan–gold nanoparticles and computational modeling of the interaction between chitosan and a lipid bilayer model. Nanomaterials 10 (12), 2340. 10.3390/nano10122340 33255714 PMC7761461

[B38] Gao Y. Zhou D. Lyu J. Xu Q. Newland B. Matyjaszewski K. (2020). Complex polymer architectures through free-radical polymerization of multivinyl monomers. Nat. Rev. Chem. 4 (4), 194–212. 10.1038/s41570-020-0170-7 37128047

[B39] Geeva Prasanth A. Sathish Kumar A. Sai Shruthi B. Subramanian S. (2019). Kinetic study and *in vitro* drug release studies of nitrendipine loaded arylamide grafted chitosan blend microspheres. Mater. Res. Express 6 (12), 125427. 10.1088/2053-1591/ab5811

[B40] Georgopoulou A. Kaliva M. Vamvakaki M. Chatzinikolaidou M. (2018). Osteogenic potential of pre-osteoblastic cells on a chitosan-graft-polycaprolactone copolymer. Mater. (Basel) 11 (4), 490. 10.3390/ma11040490 PMC5951336 29587410

[B41] Gu G. Erişen D. E. Yang K. Zhang B. Shen M. Zou J. (2022). Antibacterial and anti-inflammatory activities of chitosan/copper complex coating on medical catheters: *In vitro* and *in vivo* . J. Biomed. Mater Res. B Appl. Biomater. 110 (8), 1899–1910. 10.1002/jbm.b.35047 35253986

[B42] Guan G. Azad M. A. K. Lin Y. Kim S. W. Tian Y. Liu G. (2019). Biological effects and applications of chitosan and chito-oligosaccharides. Front. Physiol. 10, 516. 10.3389/fphys.2019.00516 31133871 PMC6514239

[B43] Guan Z. Feng Q. (2022). Chitosan and Chitooligosaccharide: The promising non-plant-derived prebiotics with multiple biological activities. Int. J. Mol. Sci. 23 (12), 6761. 10.3390/ijms23126761 35743209 PMC9223384

[B44] Gupta V. K. Pathania D. Priya B. Singha A. S. Sharma G. (2014). Microwave induced synthesis of graft copolymer of binary vinyl monomer mixtures onto delignified Grewia optiva fiber: Application in dye removal. Front. Chem. 2, 59. 10.3389/fchem.2014.00059 25157348 PMC4127470

[B45] Hamed I. Özogul F. Regenstein J. M. (2016). Industrial applications of crustacean by-products (chitin, chitosan, and chitooligosaccharides): A review. Trends Food Sci. Technol. 48, 40–50. 10.1016/j.tifs.2015.11.007

[B46] Hawthorne D. Pannala A. Sandeman S. Lloyd A. (2022). Sustained and targeted delivery of hydrophilic drug compounds: A review of existing and novel technologies from bench to bedside. J. Drug Deliv. Sci. Technol. 78, 103936. 10.1016/j.jddst.2022.103936

[B47] Hu F. Q. Wu X. L. Du Y. Z. You J. Yuan H. (2008). Cellular uptake and cytotoxicity of shell crosslinked stearic acid-grafted chitosan oligosaccharide micelles encapsulating doxorubicin. Eur. J. Pharm. Biopharm. 69 (1), 117–125. 10.1016/j.ejpb.2007.09.018 17997293

[B48] Huang C. Dong L. Zhao B. Lu Y. Huang S. Yuan Z. (2022). Anti-inflammatory hydrogel dressings and skin wound healing. Clin. Transl. Med. 12 (11), e1094. 10.1002/ctm2.1094 36354147 PMC9647861

[B49] Iacob A. T. Lupascu F. G. Apotrosoaei M. Vasincu I. M. Tauser R. G. Lupascu D. (2021). Recent biomedical approaches for chitosan based materials as drug delivery nanocarriers. Pharmaceutics 13 (4), 587. 10.3390/pharmaceutics13040587 33924046 PMC8073149

[B50] Jommanee N. Chanthad C. Manokruang K. (2018). Preparation of injectable hydrogels from temperature and pH responsive grafted chitosan with tuned gelation temperature suitable for tumor acidic environment. Carbohydr. Polym. 198, 486–494. 10.1016/j.carbpol.2018.06.099 30093026

[B51] Kaczmarek B. Nadolna K. Owczarek A. (2020). The physical and chemical properties of hydrogels based on natural polymers. Hydrogels based on natural polymers, 151–172.

[B52] Kai D. Zhang K. Liow S. S. Loh X. J. (2018). New dual functional PHB-grafted lignin copolymer: Synthesis, mechanical properties, and biocompatibility studies. ACS Appl. Bio Mater. 2 (1), 127–134. 10.1021/acsabm.8b00445 35016335

[B53] Kalia S. Sabaa M. W. Kango S. (2013). “Polymer grafting: A versatile means to modify the polysaccharides,” in Polysaccharide based graft copolymers. Editors Kalia S. Sabaa M. W. (Berlin, Heidelberg: Springer Berlin Heidelberg), 1–14.

[B54] Kaliva M. Georgopoulou A. Dragatogiannis D. A. Charitidis C. A. Chatzinikolaidou M. Vamvakaki M. (2020). Biodegradable chitosan-graft-poly(l-lactide) copolymers for bone tissue engineering. Polym. (Basel). 12 (2), 316. 10.3390/polym12020316 PMC7077469 32033024

[B55] Khalaf E. M. Abood N. A. Atta R. Z. Ramírez-Coronel A. A. Alazragi R. Parra R. M. R. (2023). Recent progressions in biomedical and pharmaceutical applications of chitosan nanoparticles: A comprehensive review. Int. J. Biol. Macromol. 231, 123354. 10.1016/j.ijbiomac.2023.123354 36681228

[B56] Kibungu C. Kondiah P. P. D. Kumar P. Choonara Y. E. (2021). This review recent advances in chitosan and alginate‐based hydrogels for wound healing application. Front. Mater. 8. 10.3389/fmats.2021.681960

[B57] Kim E. Kang M. Liu H. Cao C. Liu C. Bentley W. E. (2019). Pro- and Anti-oxidant properties of redox-active catechol-chitosan films. Front. Chem. 7, 541. 10.3389/fchem.2019.00541 31417897 PMC6682675

[B58] Krishnan A. Roy S. Menon S. (2022). Amphiphilic block copolymers: From synthesis including living polymerization methods to applications in drug delivery. Eur. Polym. J. 172, 111224. 10.1016/j.eurpolymj.2022.111224

[B59] Kumar D. Pandey J. Kumar P. (2018). Synthesis and characterization of modified chitosan via microwave route for novel antibacterial application. Int. J. Biol. Macromol. 107, 1388–1394. 10.1016/j.ijbiomac.2017.10.002 28986212

[B60] Kumar D. Raj V. Verma A. Kumar P. Pandey J. (2019). Novel binary grafted chitosan nanocarrier for sustained release of curcumin. Int. J. Biol. Macromol. 131, 184–191. 10.1016/j.ijbiomac.2019.03.008 30840864

[B61] Kumbar S. G. Aminabhavi T. M. (2003). Synthesis and characterization of modified chitosan microspheres: Effect of the grafting ratio on the controlled release of nifedipine through microspheres. J. Appl. Polym. Sci. 89 (11), 2940–2949. 10.1002/app.12386

[B62] Ladeira G. de Carvalho S. Y. B. Rocha N. A. P. Soares I. C. Cipriano D. F. Freitas J. C. C. D. (2023). Grafted chitosan nanogel with 3,4-methylenedioxycinnamic acid: Synthesis, characterization and application in the encapsulation of monoterpenes with antifungal properties. Int. J. Polym. Mater. Polym. Biomaterials, 1–12. 10.1080/00914037.2022.2163643

[B63] Lembo D. Donalisio M. Civra A. Argenziano M. Cavalli R. (2018). Nanomedicine formulations for the delivery of antiviral drugs: A promising solution for the treatment of viral infections. Expert Opin. Drug Deliv. 15 (1), 93–114. 10.1080/17425247.2017.1360863 28749739

[B64] Li J. Tang R. Zhang P. Yuan M. Li H. Yuan M. (2022). The Preparation and characterization of chitooligosaccharide polylactide polymers, and *in vitro* release of microspheres loaded with vancomycin. J. Funct. Biomater. 13 (3), 113. 10.3390/jfb13030113 35997451 PMC9397111

[B65] Li J. Wang D. Chang S.-C. Liang P.-H. Srivastava V. Guu S.-Y. (2021). Production of structurally defined chito-oligosaccharides with a single n-acetylation at their reducing end using a newly discovered chitinase from *Paenibacillus pabuli* . J. Agric. Food Chem. 69 (11), 3371–3379. 10.1021/acs.jafc.0c06804 33688734 PMC8041281

[B66] Li X. Hetjens L. Wolter N. Li H. Shi X. Pich A. (2023). Charge-reversible and biodegradable chitosan-based microgels for lysozyme-triggered release of vancomycin. J. Adv. Res. 43, 87–96. 10.1016/j.jare.2022.02.014 36585117 PMC9811367

[B67] Liu H. Wang C. Li C. Qin Y. Wang Z. Yang F. (2018). A functional chitosan-based hydrogel as a wound dressing and drug delivery system in the treatment of wound healing. RSC Adv. 8 (14), 7533–7549. 10.1039/c7ra13510f 35539132 PMC9078458

[B68] Liu J. Wang X. Bai R. Zhang N. Kan J. Jin C. (2018). Synthesis, characterization, and antioxidant activity of caffeic-acid-grafted corn starch. Starch - Stärke. 70 (1-2), 1700141. 10.1002/star.201700141

[B69] Liu S. Zhao Y. Wei H. Nie L. Ding P. Sun H. (2022). Injectable hydrogels based on silk fibroin peptide grafted hydroxypropyl chitosan and oxidized microcrystalline cellulose for scarless wound healing. Colloids Surfaces A Physicochem. Eng. Aspects 647, 129062. 10.1016/j.colsurfa.2022.129062

[B70] Lobo V. Patil A. Phatak A. Chandra N. (2010). Free radicals, antioxidants and functional foods: Impact on human health. Pharmacogn. Rev. 4 (8), 118–126. 10.4103/0973-7847.70902 22228951 PMC3249911

[B71] Logigan C.-L. Delaite C. Tiron C.-E. Peptu C. Popa M. Peptu C. A. (2022). Chitosan grafted poly (ethylene glycol) methyl ether acrylate particulate hydrogels for drug delivery applications. Gels 8 (8), 494. 10.3390/gels8080494 36005095 PMC9407074

[B72] Mahanta A. K. Maiti P. (2019). Injectable hydrogel through hydrophobic grafting on chitosan for controlled drug delivery. ACS Appl. Bio Mater. 2 (12), 5415–5426. 10.1021/acsabm.9b00733 35021540

[B73] Mahdavinia G. R. Mosallanezhad A. Soleymani M. Sabzi M. (2017). Magnetic- and pH-responsive κ-carrageenan/chitosan complexes for controlled release of methotrexate anticancer drug. Int. J. Biol. Macromol. 97, 209–217. 10.1016/j.ijbiomac.2017.01.012 28064053

[B74] Makadia H. K. Siegel S. J. (2011). Poly lactic-co-glycolic acid (PLGA) as biodegradable controlled drug delivery carrier. Polymers 3 (3), 1377–1397. 10.3390/polym3031377 22577513 PMC3347861

[B75] Manivasagan P. Bharathiraja S. Bui N. Q. Lim I. G. Oh J. (2016). Paclitaxel-loaded chitosan oligosaccharide-stabilized gold nanoparticles as novel agents for drug delivery and photoacoustic imaging of cancer cells. Int. J. Pharm. 511 (1), 367–379. 10.1016/j.ijpharm.2016.07.025 27424169

[B76] Mao S. Liu X. Xia W. (2021). Chitosan oligosaccharide-g-linalool polymer as inhibitor of hyaluronidase and collagenase activity. Int. J. Biol. Macromol. 166, 1570–1577. 10.1016/j.ijbiomac.2020.11.036 33189750

[B77] Mao S. Wang B. Yue L. Xia W. (2021). Effects of citronellol grafted chitosan oligosaccharide derivatives on regulating anti-inflammatory activity. Carbohydr. Polym. 262, 117972. 10.1016/j.carbpol.2021.117972 33838788

[B78] Michalek L. Krappitz T. Mundsinger K. Walden S. L. Barner L. Barner-Kowollik C. (2020). Mapping photochemical reactivity profiles on surfaces. J. Am. Chem. Soc. 142 (52), 21651–21655. 10.1021/jacs.0c11485 33337866

[B79] Mittal A. Singh A. Benjakul S. Prodpran T. Nilsuwan K. Huda N. (2021). Composite films based on chitosan and epigallocatechin gallate grafted chitosan: Characterization, antioxidant and antimicrobial activities. Food Hydrocoll. 111, 106384. 10.1016/j.foodhyd.2020.106384

[B80] Moretton M. A. Chiappetta D. A. Sosnik A. (2012). Cryoprotection–lyophilization and physical stabilization of rifampicin-loaded flower-like polymeric micelles. J. R. Soc. Interface 9 (68), 487–502. 10.1098/rsif.2011.0414 21865255 PMC3262430

[B81] Naikwadi A. T. Sharma B. K. Bhatt K. D. Mahanwar P. A. (2022). Gamma radiation processed polymeric materials for high performance applications: A review. Front. Chem. 10, 837111. 10.3389/fchem.2022.837111 35360545 PMC8964295

[B82] Nawrotek K. Tylman M. Rudnicka K. Balcerzak J. Kamiński K. (2016). Chitosan-based hydrogel implants enriched with calcium ions intended for peripheral nervous tissue regeneration. Carbohydr. Polym. 136, 764–771. 10.1016/j.carbpol.2015.09.105 26572411

[B83] Ngo D.-H. Kim S.-K. (2014). “Chapter Two - antioxidant effects of chitin, chitosan, and their derivatives,” in Advances in food and nutrition research. Editor Kim S.-K. (Academic Press), 73, 15–31. 10.1016/B978-0-12-800268-1.00002-0 25300540

[B84] Nwabor O. F. Singh S. Paosen S. Vongkamjan K. Voravuthikunchai S. P. (2020). Enhancement of food shelf life with polyvinyl alcohol-chitosan nanocomposite films from bioactive Eucalyptus leaf extracts. Food Biosci. 36, 100609. 10.1016/j.fbio.2020.100609

[B85] Olicón-Hernández D. R. Vázquez-Landaverde P. A. Cruz-Camarillo R. Rojas-Avelizapa L. I. (2017). Comparison of chito-oligosaccharide production from three different colloidal chitosans using the endochitonsanolytic system of Bacillus thuringiensis. Prep. Biochem. Biotechnol. 47 (2), 116–122. 10.1080/10826068.2016.1181086 27830993

[B86] Ortega A. Sánchez A. Burillo G. (2021). Binary graft of poly(n-vinylcaprolactam) and poly(acrylic acid) onto chitosan hydrogels using ionizing radiation for the retention and controlled release of therapeutic compounds. Polym. (Basel). 13 (16), 2641. 10.3390/polym13162641 PMC8397969 34451181

[B87] Ortiz‐Rodríguez T. De La Fuente‐Salcido N. Bideshi D. Salcedo‐Hernández R. Barboza‐Corona J. (2010). Generation of chitin‐derived oligosaccharides toxic to pathogenic bacteria using ChiA74, an endochitinase native to Bacillus thuringiensis. Lett. Appl. Microbiol. 51 (2), 184–190. 10.1111/j.1472-765X.2010.02876.x 20557451

[B88] Ouyang Y. Zhao J. Wang S. (2023). Multifunctional hydrogels based on chitosan, hyaluronic acid and other biological macromolecules for the treatment of inflammatory bowel disease: A review. Int. J. Biol. Macromol. 227, 505–523. 10.1016/j.ijbiomac.2022.12.032 36495992

[B89] Pagar A. D. Patil M. D. Flood D. T. Yoo T. H. Dawson P. E. Yun H. (2021). Recent advances in biocatalysis with chemical modification and expanded amino acid alphabet. Chem. Rev. 121 (10), 6173–6245. 10.1021/acs.chemrev.0c01201 33886302

[B90] Parkatzidis K. Wang H. S. Truong N. P. Anastasaki A. (2020). Recent developments and future challenges in controlled radical polymerization: A 2020 update. Chem 6 (7), 1575–1588. 10.1016/j.chempr.2020.06.014

[B91] Parvez S. Yadagiri G. Karole A. Singh O. P. Verma A. Sundar S. (2020). Recuperating biopharmaceutical aspects of amphotericin b and paromomycin using a chitosan functionalized nanocarrier via oral route for enhanced anti-leishmanial activity. Front. Cell. Infect. Microbiol. 10, 570573. 10.3389/fcimb.2020.570573 33178626 PMC7593694

[B92] Pham-Huy L. A. He H. Pham-Huy C. (2008). Free radicals, antioxidants in disease and health. Int. J. Biomed. Sci. 4 (2), 89–96. 23675073 PMC3614697

[B93] Phan T. T. Phan D. T. Cao X. T. Huynh T.-C. Oh J. (2021). Roles of chitosan in green synthesis of metal nanoparticles for biomedical applications. Nanomaterials 11 (2), 273. 10.3390/nano11020273 33494225 PMC7909772

[B94] Purohit P. Bhatt A. Mittal R. K. Abdellattif M. H. Farghaly T. A. (2022). Polymer Grafting and its chemical reactions. Front. Bioeng. Biotechnol. 10, 1044927. 10.3389/fbioe.2022.1044927 36714621 PMC9874337

[B95] Purohit V. Pieta M. Pietrasik J. Plummer C. (2022). Recent advances in the ring-opening polymerization of sulfur-containing monomers. Polym. Chem. 13, 4858–4878. 10.1039/d2py00831a

[B96] Qamruzzaman M. Ahmed F. Mondal M. I. H. (2022). An overview on starch-based sustainable hydrogels: Potential applications and aspects. J. Polym. Environ. 30 (1), 19–50. 10.1007/s10924-021-02180-9

[B97] Raizaday A. Yadav H. K. Kasina S. (2022). Chitosan and its derivatives as a potential nanobiomaterial: Drug delivery and biomedical application. Recent Trends Nanomedicine Tissue Eng., 57–94. 10.1201/9781003339236-3

[B98] Ramırez M. G. Avelizapa L. R. Avelizapa N. R. Camarillo R. C. (2004). Colloidal chitin stained with Remazol Brilliant Blue R®, a useful substrate to select chitinolytic microorganisms and to evaluate chitinases. J. Microbiol. Methods 56 (2), 213–219. 10.1016/j.mimet.2003.10.011 14744450

[B99] Rosas-García N. M. Fortuna-González J. M. Barboza-Corona J. E. (2013). Characterization of the chitinase gene in Bacillus thuringiensis Mexican isolates. Folia Microbiol. 58, 483–490. 10.1007/s12223-013-0233-y 23456349

[B100] Roy D. Semsarilar M. Guthrie J. T. Perrier S. (2009). Cellulose modification by polymer grafting: A review. Chem. Soc. Rev. 38 (7), 2046–2064. 10.1039/b808639g 19551181

[B101] Saeed R. M. Dmour I. Taha M. O. (2020). Stable Chitosan-based nanoparticles using polyphosphoric acid or hexametaphosphate for tandem ionotropic/covalent crosslinking and subsequent investigation as novel vehicles for drug delivery. Front. Bioeng. Biotechnol. 8, 4. 10.3389/fbioe.2020.00004 32039190 PMC6993129

[B102] Said M. Atassi Y. Tally M. Khatib H. (2018). Environmentally friendly chitosan-g-poly(acrylic acid-co-acrylamide)/ground basalt superabsorbent composite for agricultural applications. J. Polym. Environ. 26 (9), 3937–3948. 10.1007/s10924-018-1269-5

[B103] Sakhare M. S. Rajput H. H. (2017). Polymer grafting and applications in pharmaceutical drug delivery systems - a brief review. Asian J. Pharm. Clin. Res. 10 (6), 59–63. 10.22159/ajpcr.2017.v10i6.18072

[B104] Sanchez-Salvador J. L. Balea A. Monte M. C. Negro C. Blanco A. (2021). Chitosan grafted/cross-linked with biodegradable polymers: A review. Int. J. Biol. Macromol. 178, 325–343. 10.1016/j.ijbiomac.2021.02.200 33652051

[B105] Sang Z. Qian J. Han J. Deng X. Shen J. Li G. (2020). Comparison of three water-soluble polyphosphate tripolyphosphate, phytic acid, and sodium hexametaphosphate as crosslinking agents in chitosan nanoparticle formulation. Carbohydr. Polym. 230, 115577. 10.1016/j.carbpol.2019.115577 31887915

[B106] Santos V. P. Marques N. S. S. Maia P. C. S. V. Lima M. A. B. D. Franco L. D. O. Campos-Takaki G. M. D. (2020). Seafood waste as attractive source of chitin and chitosan production and their applications. Int. J. Mol. Sci. 21 (12), 4290. 10.3390/ijms21124290 32560250 PMC7349002

[B107] Schmitz C. González Auza L. Koberidze D. Rasche S. Fischer R. Bortesi L. (2019). Conversion of chitin to defined chitosan oligomers: Current status and future prospects. Mar. drugs 17 (8), 452. 10.3390/md17080452 31374920 PMC6723438

[B108] Sharma B. Sharma S. Jain P. (2021). Leveraging advances in chemistry to design biodegradable polymeric implants using chitosan and other biomaterials. Int. J. Biol. Macromol. 169, 414–427. 10.1016/j.ijbiomac.2020.12.112 33352152

[B109] Shokri Z. Seidi F. Saeb M. R. Jin Y. Li C. Xiao H. (2022). Elucidating the impact of enzymatic modifications on the structure, properties, and applications of cellulose, chitosan, starch and their derivatives: A review. Mater. Today Chem. 24, 100780. 10.1016/j.mtchem.2022.100780

[B110] Singh S. Nwabor O. F. Syukri D. M. Voravuthikunchai S. P. (2021). Chitosan-poly(vinyl alcohol) intelligent films fortified with anthocyanins isolated from *Clitoria ternatea* and *Carissa carandas* for monitoring beverage freshness. Int. J. Biol. Macromol. 182, 1015–1025. 10.1016/j.ijbiomac.2021.04.027 33839180

[B111] Song X. Chen Y. Zhao G. Sun H. Che H. Leng X. (2020). Effect of molecular weight of chitosan and its oligosaccharides on antitumor activities of chitosan-selenium nanoparticles. Carbohydr. Polym. 231, 115689. 10.1016/j.carbpol.2019.115689 31888818

[B112] Subramanian K. G. Vijayakumar V. (2012). Synthesis and evaluation of chitosan-graft-poly (2-hydroxyethyl methacrylate-co-itaconic acid) as a drug carrier for controlled release of tramadol hydrochloride. Saudi Pharm. J. 20 (3), 263–271. 10.1016/j.jsps.2011.09.004 23960799 PMC3744969

[B113] Sunarti T. C. Febrian M. I. Ruriani E. Yuliasih I. (2019). Some properties of chemical cross-linking biohydrogel from starch and chitosan. Int. J. Biomaterials 2019, 1–6. 10.1155/2019/1542128 PMC6425316 30949205

[B114] Tachaboonyakiat W. (2017). Antimicrobial applications of chitosan. Chitosan based Biomater. Vol. 2, 245–274. Elsevier.

[B115] Tan L. S. Tan H. L. Deekonda K. Wong Y. Y. Muniyandy S. Hashim K. (2021). Fabrication of radiation cross-linked diclofenac sodium loaded carboxymethyl sago pulp/chitosan hydrogel for enteric and sustained drug delivery. Carbohydr. Polym. Technol. Appl. 2, 100084. 10.1016/j.carpta.2021.100084

[B116] Tan W. Zhang J. Mi Y. Li Q. Guo Z. (2022). Synthesis and characterization of α-lipoic acid grafted chitosan derivatives with antioxidant activity. React. Funct. Polym. 172, 105205. 10.1016/j.reactfunctpolym.2022.105205

[B117] Tschan M. J.-L. Gauvin R. M. Thomas C. M. (2021). Controlling polymer stereochemistry in ring-opening polymerization: A decade of advances shaping the future of biodegradable polyesters. Chem. Soc. Rev. 50 (24), 13587–13608. 10.1039/d1cs00356a 34786575

[B118] Vega-Hernández M. Á. Cano-Díaz G. S. Vivaldo-Lima E. Rosas-Aburto A. Hernández-Luna M. G. Martinez A. (2021). A Review on the synthesis, characterization, and modeling of polymer grafting. Processes 9 (2), 375. 10.3390/pr9020375

[B119] Wan A. Xu Q. Sun Y. Li H. (2013). Antioxidant Activity of high molecular weight chitosan and n,o-quaternized chitosans. J. Agric. Food Chem. 61 (28), 6921–6928. 10.1021/jf402242e 23706102

[B120] Wang W. Xue C. Mao X. (2020). Chitosan: Structural modification, biological activity and application. Int. J. Biol. Macromol. 164, 4532–4546. 10.1016/j.ijbiomac.2020.09.042 32941908

[B121] Wang X. Lou T. Zhao W. Song G. (2016). Preparation of pure chitosan film using ternary solvents and its super absorbency. Carbohydr. Polym. 153, 253–257. 10.1016/j.carbpol.2016.07.081 27561494

[B122] Ways T. M. M. Filippov S. K. Maji S. Glassner M. Cegłowski M. Hoogenboom R. (2022). Mucus-penetrating nanoparticles based on chitosan grafted with various non-ionic polymers: Synthesis, structural characterisation and diffusion studies. J. Colloid Interface Sci. 626, 251–264. 10.1016/j.jcis.2022.06.126 35797869

[B123] Wong T. W. (2009). Chitosan and its use in design of insulin delivery system. Recent Pat. drug Deliv. formulation 3 (1), 8–25. 10.2174/187221109787158346 19149726

[B124] Yang J. Chen Y. Zhao L. Zhang J. Luo H. (2022). Constructions and properties of physically cross-linked hydrogels based on natural polymers. Polym. Rev., 1–39. 10.1080/15583724.2022.2137525

[B125] Yang X. Lan W. Lu M. Wang Z. Xie J. (2022). Characterization of different phenolic acids grafted chitosan and their application for Japanese sea bass (*Lateolabrax japonicus*) fillets preservation. LWT 170, 114072. 10.1016/j.lwt.2022.114072

[B126] Yilmaz Atay H. (2019). “Antibacterial activity of chitosan-based systems,” in Functional chitosan: drug delivery and biomedical applications. Editors jana s. jana s. (Singapore: Springer Singapore), 457–489.

[B127] Youn D. K. No H. K. Prinyawiwatkul W. (2013). Preparation and characteristics of squid pen β-chitin prepared under optimal deproteinisation and demineralisation condition. Int. J. Food Sci. Technol. 48 (3), 571–577. 10.1111/ijfs.12001

[B128] Zhang P. Cao M. (2014). Preparation of a novel organo-soluble chitosan grafted polycaprolactone copolymer for drug delivery. Int. J. Biol. Macromol. 65, 21–27. 10.1016/j.ijbiomac.2014.01.009 24418345

[B129] Zheng M. Zhang C. Zhou Y. Lu Z. Zhao H. Bie X. (2018). Preparation of gallic acid-grafted chitosan using recombinant bacterial laccase and its application in chilled meat preservation. Front. Microbiol. 9, 1729. 10.3389/fmicb.2018.01729 30123192 PMC6085427

[B130] Zhuang S. Yin Y. Wang J. (2018). Removal of cobalt ions from aqueous solution using chitosan grafted with maleic acid by gamma radiation. Nucl. Eng. Technol. 50 (1), 211–215. 10.1016/j.net.2017.11.007

